# Substance abuse and susceptibility to false memory formation: a systematic review and meta-analysis

**DOI:** 10.3389/fpsyg.2023.1176564

**Published:** 2023-05-05

**Authors:** Tânia Caetano, Maria Salomé Pinho, Eduardo Ramadas, Jessica Lopes, Timóteo Areosa, Daniela Ferreira, Maria dos Anjos Dixe

**Affiliations:** ^1^Faculty of Psychology and Educational Sciences of University of Coimbra, Center for Research in Neuropsychology and Cognitive and Behavioral Intervention (CINEICC), University of Coimbra, Coimbra, Portugal; ^2^Faculty of Psychology and Educational Sciences of University of Coimbra, Neuropsychological Assessment and Ageing Processes (NAAP), University of Coimbra, Coimbra, Portugal; ^3^Center for Innovative Care and Health Technology (ciTechCare), Polytechnic of Leiria, Leiria, Portugal; ^4^VillaRamadas International Treatment Centre, Research and Innovation Department, Leiria, Portugal

**Keywords:** confabulation, false memory, false recognition, false recall, substance abuse, systematic review, meta-analysis

## Abstract

**Background:**

Substance abuse has an impact on various cognitive domains, including memory. Even though this impact has been extensively examined across different subdomains, false memory has been sparsely studied. This systematic review and meta-analysis seek to synthesize the current scientific data concerning false memory formation in individuals with a history of substance abuse.

**Methods:**

PubMed, Scopus, the Cochrane Library, Web of Science, and PsycINFO were searched to identify all experimental and observational studies in English, Portuguese, and Spanish. Studies were then examined by four independent reviewers and, if they met the inclusion criteria, assessed for their quality. The Cochrane Risk of Bias Tool for randomized controlled trials (RCT) and the Joanna Briggs Institute (JBI) critical appraisal checklists for quasi-experimental and analytic cross-sectional studies were used to assess the risk of bias.

**Results:**

From the 443 screened studies, 27 (and two more from other sources) were considered eligible for full-text review. A final 18 studies were included in the present review. Of these, 10 were conducted with alcoholics or heavy drinkers, four focused on ecstasy/polydrug users, three were done with cannabis users and one focused on methadone maintenance patients with current cocaine dependence. Regarding false memory type, 15 studies focused on false recognition/recall, and three on provoked confabulation.

**Conclusions:**

None but one of the studies considering false recognition/recall of critical lures found any significant differences between individuals with a history of substance abuse and healthy controls. However, most of the studies taking into account false recognition/recall of related and unrelated events found that individuals with a history of substance abuse showed significantly higher rates of false memories than controls. Future research should continue to consider different types of false memories as well as their potential association with relevant clinical variables.

**Systematic review registration:**

https://www.crd.york.ac.uk/prospero/display_record.php?RecordID=266503, identifier: CRD42021266503.

## Highlights

- More research is needed on false memories and substance abuse.
- False recognition/recall of critical lures does not seem to show significant differences.
- False recognition/recall (related/unrelated items) increases false memories susceptibility.

## 1. Introduction

Substance use can negatively influence one's memory (Kloft et al., 2021). According to the Diagnostic and Statistical Manual of Mental Disorders [American Psychiatric Association (APA), 2014], substance use disorders are characterized by a set of cognitive, behavioral and physiological symptoms and their diagnosis are based on behavioral patterns. These behavioral patterns can take on a relapsing and chronic presentation because of the alterations they can cause to brain circuits [American Psychiatric Association (APA), 2014]. Some of these circuits regulate memory and studies have shown them to suffer structural changes in individuals with chronic use of alcohol (Sullivan and Pfefferbaum, 2013) or illicit substances (Cadet et al., 2014).

Different subdomains of memory have been studied in this population. Among other areas, deficits have been found in verbal memory (e.g., Ardila et al., 1991; Pope et al., 2001; Scott et al., 2007; Woicik et al., 2009; for cannabis, cocaine, and methamphetamine users), visual memory (e.g., Strickland et al., 1993; Gillen et al., 1998; Bolla et al., 2002; for cannabis, and cocaine users), working memory (e.g., Rendell et al., 2009; Hanson et al., 2010; Meier et al., 2012; Vonmoos et al., 2013; for cannabis, cocaine, and methamphetamine users), and memory recall (e.g., Thomasius et al., 2006; Battisti et al., 2010; for cannabis, and ecstasy users). Alcohol abuse has also been linked to significant memory deficits, namely verbal episodic (e.g., Chanraud et al., 2009) and long-term memory (e.g., Defranco et al., 1985). In its extreme, chronic alcohol abuse can lead to symptoms of amnesia, for example through the development of Korsakoff syndrome (e.g., Arts et al., 2017).

Even though the impact of substance abuse and addiction on memory has been widely studied, there are some phenomena in this cognitive domain that seem to have received insufficient interest. This is the case with the impact of substance abuse on the formation of false memories (Kloft et al., 2021).

### 1.1. False memories

Formation of memory can be divided into three distinct stages: encoding, consolidation, and retrieval. Each stage carries with it specific processes that are exposed to interference making the creation of a perfect memory an impossibility. As such, memories are always flawed reestablishments of reality (Straube, 2012; Kloft et al., 2021). A memory is considered to be false when it entails the recollection of an event that never happened or the distortion of one that did happen, with the presence of details that do not correspond to reality (Roediger and McDermott, 1995).

Several theories have been proposed to explain the formation of false memories. The fluency-misattribution perspective (Jacoby et al., 1989), suggests that the sense of familiarity that an individual experiences when having a false memory results from an unconscious attribution of the processing fluency (ease of information processing) to the past (i.e., incorrect source). On the other hand, the source-monitoring framework emphasizes the distinction between a memory's content and its source (Johnson et al., 1971; Johnson and Raye, 1981). According to this view, false memories are created when an individual wrongly attributes a memory to an external source (external stimuli), when it was internally produced (e.g., thought; Johnson, 1977). This describes a failure in reality monitoring. Roediger et al. (2001) build on this approach to explain the false recognition phenomenon seen in the Deese-Roediger-McDermott (DRM) paradigm (Deese, 1959; Roediger and McDermott, 1995) and develop the activation-monitoring account. It considers that during the study phase of the lists of words associated with another no presented word of the DRM paradigm, an individual may internally activate representations of associated but not presented words (including critical lure words). This can happen in a conscious (elaborative processing) or unconscious (spread activation inside an associative network) manner. Misattribution of the internal activation of the words to the outside world (source-monitoring error) can lead to the creation of false memories (e.g., McDermott and Watson, 2001). Finally, the fuzzy trace theory (e.g., Brainerd and Reyna, 2002, 2004) proposes that, when a memory is created, two distinct traces were established: verbatim and gist traces. The verbatim trace relates to the surface characteristics of the external stimuli while the gist trace relates to its theme. The first is the major driver of veridical memory, and the second serves as the base for false memories, partially because it tends to persist longer in time (Steffens and Mecklenbräuker, 2007).

False memories have been studied in various contexts and with recourse to very diverse methods. There is still considerable disagreement concerning the different types of false memories, whether they are essentially the same or significantly distinct phenomena and, as such, whether they share underlying mechanisms. The lack of consensus is a testament to the complexity of memory and signals the need for a careful discussion of this topic. Nonetheless, in the interest of clarity, throughout this review we will use similar distinctions to those proposed by Kopelman (1999) and consider the following type of false memories: false recognition/false recall, provoked/momentary confabulation, and spontaneous confabulation.

#### 1.1.1. False recognition/false recall

False recognition has propensity to appear when new items, which are conceptually or perceptually associated with previously presented items, are wrongly perceived as being old (Pierce et al., 2005; Brady et al., 2015). In the case of false recall, the new items may wrongly be retrieved in a trial to reproduce the presented material. There are two main interpretations of this phenomenon that were described above. The first draws on the activation monitoring account (e.g., Roediger et al., 2001), and considers false recognition/recall to result from an initial activation of related events at the time of the study, and a subsequent failure to discriminate between internally and externally activated events at the time of the test. The second interpretation, considers this type of false memory to be gist- or schema-based and to constitute an adaptive memory distortion (Gutchess and Schacter, 2012; Brady et al., 2015). Gist-based representations are thought to reflect the retention of common themes between presented objects and, consequently, allow for quicker recognition of new items that are consistent with the relevant theme. Gist-based false recognition is then considered an adaptive cognitive process that can translate to increased performance in certain tasks. For example, the propensity for gist-based false recognition is correlated to creativity (Dewhurst et al., 2011).

Measures of false recognition or false recall can be found in several memory tasks but the most used method to study this type of false memory is the DRM paradigm (Deese, 1959; Roediger and McDermott, 1995). The DRM paradigm starts with the presentation of a list of semantically related words (e.g., bed, rest, dream) at encoding. After a delay, individuals are asked either to reproduce the studied words (free recall test) or to identify them from a new list (recognition test) where both the previously presented words and the critical lure words (e.g., sleep; related words not previously shown) are included (Pardilla-Delgado and Payne, 2017).

Neuroimaging studies tend to indicate an overlap between brain regions activated during true and false recognition (Johnson et al., 1997; Cabeza et al., 2001; Garoff-Eaton et al., 2006). These results have been considered as further evidence for the conception of false recognition as an adaptive memory distortion (Schacter et al., 2011). Garoff-Eaton et al. (2006) found both identical true and related false recognition associated with activation of a wide range of brain regions (prefrontal, lateral, medial temporal, parietal, and occipital cortices).

#### 1.1.2. Confabulation

While false recall/recognition can be experienced by everyone and is even considered potentially adaptive, confabulation has been mostly associated with clinical populations (Hirstein, 2009). It was a term first described by Korsakoff (1887; 1889a; 1889b as cited in Kopelman et al., 2009) about amnesic patients, and has been historically linked to brain damage and neurological syndromes (especially those characterized by memory loss). In 1901, Bonhoeffer (as cited in Nahum et al., 2012) distinguished between two types of confabulations: spontaneous confabulation, associated with dream-like ideas; and confabulation of embarrassment (“momentary confabulation”). The latter refers to memories that appear to have been fabricated to compensate for memory loss or a “gap” in memory. One's memory void is filled with content from real memories but there are temporally displaced by the individual (Benson et al., 1996). They are often associated with memory-related diseases (Rensen et al., 2017), but they can also be experienced by healthy individuals. Similarly, Berlyne (1972), considered two different types of confabulation: “fantastic” confabulation, and “momentary” confabulation. For Berlyne (1972), “momentary” confabulations were rooted in real memory and autobiographic content. They were also a result of questioning and, as such, could be provoked. This led Kopelman (1987), to later revise the terminology, defining the terms most used today: provoked instead of momentary, spontaneous instead of fantastic.

Various methods are used to evaluate provoked confabulations in adults. These types of false memories can be induced through the “misinformation effect”, which consists of the exposure to interfering and false information after having witnessed an event (Hirstein, 2009). They can be studied with recourse to psychometric instruments, namely the Dalla Barba Confabulation Battery (DBCB; Dalla Barba, 1993), the Provoked Confabulation Test (PCT; Cooper et al., 2006), and suggestibility tests such as the Gudjonsson Suggestibility Scale (GSS; Gudjonsson, 1987). Regarding the context, suggestibility tests tend to be used to assess interrogative suggestibility and the production of false memories in a forensic setting, while the other cited instruments appear more often associated with the assessment of general and clinical samples. The DBCB was purposefully created to quantify and qualify confabulations in confabulating patients. It is composed of 165 questions divided across eleven different domains, including personal, linguistic, and recent general semantic memory (Dalla Barba et al., 2018). While by definition (since it is based on questioning), the DBCB assesses provoked confabulations, clinical observation seems to indicate that those who confabulate at the battery, tend to also confabulate spontaneously (Dalla Barba et al., 2018). Even though for practical reasons research has been mostly focused on provoked confabulations, recently an observational scale has been developed to evaluate not only provoked but also spontaneous confabulations. According to Rensen et al. (2015), ratings in the Nijmegen-Venray Confabulation List-20 (NVCL) are related to ratings on the DBCB and the PCT.

It is still not clear which brain regions and cognitive deficits are directly involved in the formation of confabulations (Turner et al., 2008). The most studied confabulation presentation is that believed to be caused by damage in the frontal lobe (Turner et al., 2008; Hirstein, 2009), namely resulting from Korsakoff's Syndrome (Berlyne, 1972; Kopelman, 1987; Barba et al., 1990; Benson et al., 1996; Kopelman et al., 1997), head injury (Damasio et al., 1985; Baddeley and Wilson, 1988; Moscovitch and Melo, 1997; Box et al., 1999), and frontotemporal dementia (Moscovitch and Melo, 1997; Nedjam et al., 2000). Given the type of neurological conditions that appear most often associated with confabulations, many believe that frontal lobe damage may be critical to its formation (Hirstein, 2009). In a review conducted by Gilboa and Moscovitch (2002), 81% of confabulators presented damage in the pre-frontal cortex. Particularly, they observed that damage to the orbitofrontal and ventromedial cortices was the most common in association with the occurrence of confabulations. Schnider (2003), Schnider et al. (1996), and Schnider and Ptak (1999), also found the orbitofrontal cortex as a possible critical site of injury regarding spontaneous confabulation. Despite the high prevalence of frontal lobe damage in confabulators, some studies suggest that pathology in this brain area may not be necessary or sufficient for the occurrence of confabulation. Reports indicate that patients can present confabulations without associated frontal executive dysfunction or structural pathology (Nedjam et al., 2000). Moreover, frontal lobe impairment appears not to distinguish between amnesiacs who confabulate from those who do not (Schnider, 2003). According to Gündogar and Demirci (2007), the provoked confabulations have no specific brain location. The same authors mentioned that only spontaneous confabulations arise from injuries in the posterior orbitofrontal cortex and basal forebrain.

#### 1.1.3. False memories and substance abuse

The impact of substance abuse on false memory formation is not clear. There seem to be few studies looking into provoked and spontaneous confabulations in this population, and those focusing on false recognition/false recall have shown mixed results. Using the DRM paradigm, Rocha and Albuquerque (2003), found that alcoholics were not more likely to falsely recognize critical lures, but they also presented significant differences compared to controls concerning other intrusion errors. Similarly, Kloft et al. (2019), found that cannabis users presented similar false memory rates for critical lures to those of controls, but significantly higher false recognition rates for unrelated items. Riba et al. (2015), however, using functional magnetic resonance imaging, showed that cannabis users did present increased susceptibility to false memories by failing to correctly identify critical lures as events that never occurred.

Beyond contradictory results regarding the more well-accepted measure of false recognition/false recall (i.e., false recognition/false recall of critical lures), these and other studies (Reich et al., 2004; Cuttler et al., 2021) suggest an apparent distinction between this measure and other types of intrusion errors and false recognition indicators. Even though there is no obvious consensus on what these indicators are measuring—just as there is no consensus on the association between false recognition measures and real-life false memories formation—they still appear to be relevant for a broader understanding of the impact of substance abuse on memory in general, and on the susceptibility to this phenomenon in particular. Because, to our knowledge, this is the first systematic review and meta-analysis looking into the susceptibility to false memory formation of individuals with a history of substance abuse, we will consider studies that use any type of false memory measure, including false recognition/false recall of events other than critical lures. Provided that the necessary information is available in the selected studies, we intend to answer the following research questions:

I- Are individuals with a history of substance abuse more susceptible to false memory formation when compared to healthy controls? If so, to what type of false memories are they more prone to?

II- How does the susceptibility to the formation of false memories in individuals with a history of substance abuse compared to that of individuals with neurological conditions?

III- Are there any real-world implications for increased susceptibility to the formation of false memories in individuals with a history of substance abuse? If so, what are they?

## 2. Materials and methods

### 2.1. Protocol registration

The protocol outlining the goals and scope of the present systematic literature review and meta-analysis was registered and published in the International Prospective Register of Systematic Reviews (PROSPERO) on the 7th of August of 2021 (Caetano et al., 2021). The protocol is available from: https://www.crd.york.ac.uk/prospero/display_record.php?ID=CRD42021266503.

### 2.2. Search strategy

The Population, Intervention, Comparison, and Outcome (PICO) strategy of the Joanna Briggs Institute (JBI; Aromataris and Munn, 2017) served as the basis for this systematic review. The main goal was to understand if individuals with a history of substance abuse (with or without a current diagnosis of substance use disorder) show a higher susceptibility to the formation of false memories. Publication date and period were not restricted to include all possibly relevant studies, and all articles, published and unpublished, written in English, Portuguese, and Spanish were considered.

At the first moment, a general search was conducted in the Cochrane Database of Systematic Reviews, the JBI Database of Systematic Reviews and Implementation Reports, MEDLINE, and PROSPERO to confirm the absence of systematic reviews with identical objectives. Following that, an exhaustive but limited search was performed in the databases PubMed, Scopus, the Cochrane Library, Web of Science, and PsycINFO. The titles of the articles found were examined for relevant search terms. The search terms originated from DeCS^®^ and Medical Subject Headings (MeSH Browser^®^) and were selected taking into account the PICO strategy. They were combined with Boolean operators for a more focused and productive search. Below are the keywords used in the search: *Substance-Related Disorders, Substance-Use Disorders, Addiction Medicine, Addiction Treatment, Drug Abusers, Drug Abuse, Drug Addiction, Alcoholism, Alcohol Abuse, Alcohol Dependence, Chronic Alcoholism, Alcohol Addiction, Alcohol-Related Disorder, Substance Abuse, Drug Dependence, Substance Addiction, False Memory, False Memories, DRM Paradigm, Confabulation, Provoked Confabulation, Spontaneous Confabulation*. The Boolean operators were arranged as follows: (*Substance-Related Disorders* OR *Substance-Use Disorders* OR *Addiction Medicine* OR *Addiction Treatment* OR *Drug Abusers* OR *Drug Abuse* OR *Drug Addiction* OR *Alcoholism* OR *Alcohol Abuse* OR *Alcohol Dependence* OR *Chronic Alcoholism* OR *Alcohol addiction* OR *Alcohol-Related Disorder* OR *Substance Abuse* OR *Drug Dependence* OR *Substance Addiction*) AND (*False Memory* OR *False Memories* OR *DRM Paradigm* OR *Confabulation* OR *Provoked Confabulation* OR *Spontaneous Confabulation*). The final database search was conducted in July 2021. Finally, the references of all selected studies were searched for relevant studies that did not appear in the initial search.

### 2.3. Study selection and data extraction

Titles and abstracts from the retrieved articles were screened by four independent reviewers to identify all experimental, cross-sectional, case-control, or cohort studies that included: adult participants (age of 18 years or above) with a history of substance abuse (with or without a diagnosis of substance use disorder) and without a neurological condition (e.g., Korsakoff's syndrome); Exposure to at least one procedure aimed at inducing/evaluating or with a measure of false memory; With or without a comparison group (healthy control or individuals with a neurological disorder such as Korsakoff's syndrome); Outcomes of either false recognition/false recall of critical lures, false recognition/false recall of related and/or unrelated items or provoked confabulation.

Studies selected by either of the reviewers were retrieved for full-text review and assessed for eligibility for inclusion by the same four reviewers. When the four reviewers were not in agreement regarding the inclusion of a study, a fifth reviewer intervened in resolving the conflict. Articles found while handsearching underwent the same selection process. Both publication records retrieved during the review process and reasons for exclusion (when applicable) were stored in an electronic database.

Data were extracted from the included studies to assess study quality and allow for data synthesis. We extracted the year when the study was published, the country where it was conducted, the studied population/abused substance, and the details of the study methodology (e.g., study design, existence or not of comparison groups, type of comparison groups, false memory measures), the results (e.g., age mean, gender distribution, false memory measure mean and standard deviation), and limitations. Authors were not contacted for additional data not provided in the included articles. Data extraction was conducted by four reviewers and checked by another reviewer.

### 2.4. Assessment of study quality

All the included studies were independently assessed by two reviewers.

The Joanna Briggs Institute (JBI; Aromataris and Munn, 2017) critical appraisal checklist was used for quasi-experimental studies, and regarding experimental studies, the Cochrane Risk of Bias Tool (Higgins et al., 2011) was used for randomized controlled trials (RCT).

For observational studies, the JBI critical appraisal checklists (Aromataris and Munn, 2017) for analytical cross-sectional studies were used.

The Cochrane Risk of Bias Tool (Higgins et al., 2011) considers six bias domains (selection, performance, detection, attrition, reporting, and other biases), and classifies studies as presenting “unclear risk,” “low risk,” or “high risk” in each of the domains. In turn, the Joanna Briggs Institute (JBI, Aromataris and Munn, 2017) critical appraisal checklist for quasi-experimental studies considers nine yes/no questions (with the possibility to mark it as unclear or not applicable) on the extent to which each study tried to address the possibility of bias. Similarly, the JBI critical appraisal checklist (Aromataris and Munn, 2017) for analytic cross-sectional studies considers eight yes/no questions (with the possibility to mark it as unclear or not applicable) that likewise aim to evaluate the way each study sought to address possible biases.

### 2.5. Data synthesis

All the analyses were conducted using the software Review Manager 5.4.1. Where the data was available, the effect size was calculated for each of the false memory outcomes considered in the review, including false recognition of critical lures, false recognition of unrelated items, and other intrusion errors. Considering the high variability in study characteristics, the chosen effect measure was the standardized mean difference, and the effect size analyses were done using a random effects model. Moreover, when possible, subgroup analyses were performed according to false memory measures. Finally, between-study heterogeneity was assessed using standard χ^2^ tests and the I^2^ statistic. Heterogeneity is generally categorized as 25% (low), 50% (moderate), and 75% (high) (Higgins et al., 2003).

## 3. Results

### 3.1. Literature search/study selection process

**Figure 1:**
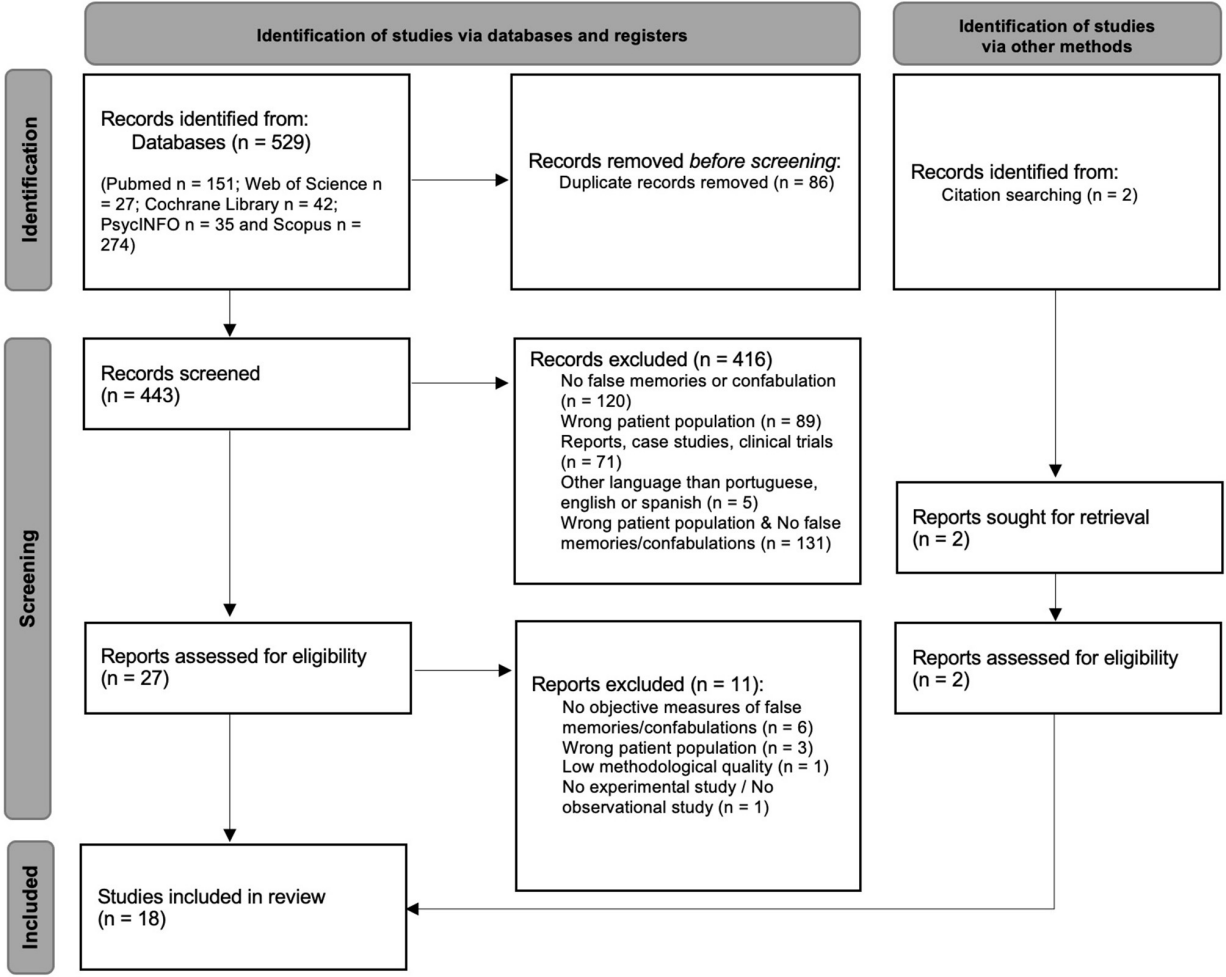
Preferred reporting items for systematic reviews and meta-analyses (PRISMA) flow diagram.

The Preferred Reporting Items for Systematic Reviews and Meta-Analyses (PRISMA) flowchart (see Figure 1) presents the study selection process for this review.

The databases search identified 529 articles (PubMed *n* = 151; Web of Science *n* = 27; Cochrane Library *n* = 42; PsycINFO *n* = 35 and Scopus *n* = 274), 86 of which were removed for being duplicated records. A total of 443 articles were screened for the established inclusion criteria, and 416 were excluded for failing to meet them. Of these 416 articles, 131 neither targeted the intended population nor presented a measure of false memory, 120 did not present a measure of false memory, 89 did not target the intended population, 71 were reports, case studies, or clinical trials, and five were published in a language other than English, Portuguese, or Spanish. The remaining 27 articles, along with two others found through citation searching, were assessed for eligibility with recourse to a full-text review. From those, 11 were excluded: six for failing to use an objective false memory measure; three for not targeting the intended population; one for not being either an experimental or observational study (i.e., scale validation study); and one for not meeting the minimal methodological quality (i.e. [selection bias, it is not an experimental study – as the title indicates, there is no randomization for participants, participants were not exposed to any procedure related with inducing false memories, which also did not allow for measurements before and after the intervention, and it was not clear what is the “cause” and what is the “effect” of the study]). Finally, 18 studies met all inclusion and exclusion criteria and were included in the systematic review, and of these only five were included in the meta-analysis.

### 3.2. Participant characteristics

In Table 1, a summary of the subject characteristics for each of the 18 included studies in the systematic review is presented.

**Table 1:** Subject characteristics.

**References**	**Country**	**Sample**	**Substance**	***n***	**Age (M ±SD), years**	**Gender (% male)**
Kramer et al. (1989)	United States	Alcoholics	Alcohol	65 in total: Younger alcoholics (*n =* 18) Older alcoholics (*n =* 16) Younger control group (n =16) Older controls (*n =* 16)	37.2 ± 6.7 in younger alcoholics 59.2 ± 3.8 in older alcoholics 33.0 ± 7.7 in younger control group 64.7 ± 5.4 in older controls	NP
Welch et al. (1997)	United States	Alcoholic subjetcs	Alcohol	162 in total: Alcohol control (n = 27) Alcohol brain-damaged (n = 30) Comparison groups: Temporal Lobe Epilepsy pre-surgery (n = 26) Temporal Lobe Epilepsy post-surgery (n = 26) Parkinson (n = 27) Neurotoxic exposure (n = 26)	51 ± 2.6 in alcohol control group; 62 ± 2.4 in alcohol brain-damaged group; Comparison groups: 32 ± 1.5 in Temporal Lobe Epilepsy pre-surgery group; 33 ± 1.5 in Temporal Lobe Epilepsy post-surgery group; 67 ± 2.1 in Parkinson group; 53 ± 2.1 Neurotoxic Exposure group.	NP
Gudjonsson et al. (2000)	Iceland	Alcoholic patients	Alcohol	75	Male: 43.0 ± 13.1 Female: 36.2 ± 11.5	± 77%
Fox et al. (2001)	United Kingdom	Regular ecstasy users	Ecstasy	42 in total: Polydrug controls (*n =* 14) Short-term Ecstasy group (*n =* 14) Long-term Ecstasy group (*n =* 14)	29.1 ± 10.9 in polydrug controls 26.4 ± 5.9 in short-term Ecstasy group 30.7 ± 4.1 in long-term Ecstasy group	± 52%
Rocha and Albuquerque (2003)	Portugal	Alcoholics	Alcohol	Experimental group (EG; *n =* 30) Healthy comparator group (CG; *n =* 23)	42.3 ± 8.5 in experimental group 42.4 ± 8.7 in control group	100%
Gudjonsson et al. (2004)	Iceland	Alcoholics	Alcohol	393	36.5 ± 13.6	71%
Reich et al. (2004)	United States	Undergraduate psychology students (heavy drinkers)	Alcohol	Alcohol expectancy (*n =* 287): Non drinker (*n =* 82) Lighter drinker (*n =* 109) Heavier drinker (*n =* 96) and Control sample (*n =* 152)	NP	± 23%
Schilt et al. (2008)	Netherlands	Poly-substance users	Ecstasy Cannabis Amphetamines Cocaine Alcohol Tobacco	67	23.5 ± 3.9	± 60%
Thoma et al. (2008)	Germany	Hospitalized alcohol-dependent detoxified patients	Alcohol	Alcohol-dependent patient group (*n =* 19) Healthy controls (*n =* 20)	45.6 ± 8.0 in alcohol-dependent patient group (ALC) 43.8 ± 11.5 in healthy controls (HC)	± 72%
Maurage et al. (2011)	Belgium	Detoxified alcoholic individuals	Alcohol	Alcoholics (n =20) Controls (*n =* 20)	50.25 ± 11.79 in alcoholic group 47.75 ± 9.73 in control group	55% in alcoholic group
Gallagher et al. (2012)	United Kingdom	University students ecstasy/polydrug users	Ecstasy and other illicit substances	Ecstasy/polydrug users (*n =* 44) Non-users (*n =* 48)	22.50 ± 2.58 in ecstasy users 20.96 ± 2.25 in non-users	NP
Henry et al. (2012)	United States	Methadone maintenance patients (MMP) with current cocaine dependence	Methadone, Cocaine	MMP with current cocaine dependence (MMP/CD+), *n =* 53 MMP without current cocaine dependence (MMP/CD–), *n =* 24	42.2 ± 0.9 in MMP/CD+ 47.0 ± 1.7 in MMP/CD–	MMP/CD+: ± 43%; MMP/CD–: ± 37%
Klein et al. (2013)	United States	Patients recently admitted to residential alcohol treatment	Alcohol	100	42.72 ± 11.95	60%
Fisk et al. (2014)	United Kingdom	Ecstasy/polydrug users	Ecstasy and other illicit substances	Ecstasy/polydrug users (*n =* 72) Non ecstasy users (*n =* 75)	22.59 ± 2.52 in male ecstasy users 21.60 ± 2.10 in female ecstasy users 21.19 ± 1.82 in male non-users 20.48 ± 2.27 in female non-users	Ecstasy users/polydrug users: ± 51%; Non-users: ± 36%
Riba et al. (2015)	Spain	Cannabis users	Cannabis	Heavy cannabis users (*n =* 16) Healthy controls (*n =* 16)	NP	NP
Brion et al. (2017)	Belgium	Patients with Korsakoff syndrome and Alcohol-dependent individuals	Alcohol	Korsakoff group (KS; *n =* 19) Alcoholic dependence (ALC; *n =* 19) Healthy control participants (CP; *n =* 19)	54.84 ± 8.00 in KS group 52.37 ± 6.15 in ALC group 52.58 ± 5.43 in CP	KS: ± 53% ALC: ± 47% CP: ± 53%
Kloft et al. (2019)	Netherlands	Cannabis users	Cannabis	Cannabis intoxication group (*n =* 53) Cannabis sober group (*n =* 50) Control group (*n =* 53)	21.6 ± 2.5 in cannabis intoxication group 21.1 ± 3.1 in cannabis sober group 22.5 ± 2.8 in control group	Cannabis intoxication: ± 87% Cannabis sober: 86% Control: ± 38%
Cuttler et al. (2021)	United States	Cannabis users	Cannabis	Cannabis users (*n =* 80): Sober (*n =* 20), Tetrahydrocannabinol (THC) (*n =* 20), THC + Cannabidiol (CBD) (*n =* 20) and Concentrates group (*n =* 20)	Total: 23,87 ± 5,67 Sober: 25.25 ± 7.37 THC flower: 23.50 ± 5.61 THC + CBD flower: 22.75 ± 4.66 Concentrates group: 24.00 ± 4.77	Total: 56.3% Sober: 50% THC flower: 55% THC + CBD flower: 70% Concentrates group: 50%

NP, not provided.

### 3.3. Date

The studies were published between 1989 and 2021, with the following distribution per publication year: one study each year in 2021 (Cuttler et al., 2021), 2019 (Kloft et al., 2019), 2017 (Brion et al., 2017), 2015 (Riba et al., 2015), 2014 (Fisk et al., 2014), 2013 (Klein et al., 2013), 2011 (Maurage et al., 2011), 2003 (Rocha and Albuquerque, 2003), 2001 (Fox et al., 2001), 2000 (Gudjonsson et al., 2000), 1997 (Welch et al., 1997), and 1989 (Kramer et al., 1989); and two studies each year; in 2012 (Gallagher et al., 2012; Henry et al., 2012), 2008 (Schilt et al., 2008; Thoma et al., 2008), and 2004 (Gudjonsson et al., 2004; Reich et al., 2004).

### 3.4. Country

Six of the 18 studies were conducted in the United States (Kramer et al., 1989; Welch et al., 1997; Reich et al., 2004; Henry et al., 2012; Klein et al., 2013; Cuttler et al., 2021), three in the United Kingdom (Fox et al., 2001; Gallagher et al., 2012; Fisk et al., 2014), two in Iceland (Gudjonsson et al., 2000, 2004), two in the Netherlands (Schilt et al., 2008; Kloft et al., 2019), and another two in Belgium (Maurage et al., 2011; Brion et al., 2017). The remaining three studies were conducted in the following countries: Portugal (Rocha and Albuquerque, 2003), Germany (Thoma et al., 2008), and Spain (Riba et al., 2015).

### 3.5. Age and gender

Three of the 10 studies looking into alcohol, presented a mean age for the experimental group above 50 years-old (Welch et al., 1997; Maurage et al., 2011; Brion et al., 2017), with two of these (Welch et al., 1997; Brion et al., 2017) having some type of neurological condition as a comparison. Another three studies presented a mean age between 40 and 50 years (Rocha and Albuquerque, 2003; Thoma et al., 2008; Klein et al., 2013). The study by Henry et al. (2012), which focused on methadone maintenance patients with and without current cocaine dependence, also presented a mean age between 40 and 50 years for both groups. From the studies focusing on alcohol, two other had participants with an overall age mean between 30 and 40 years of age (Gudjonsson et al., 2000, 2004), but one of the studies (Gudjonsson et al., 2000) chose to present the mean age of males and females separately (Male: 43.0 ± 13.1; Female: 36.2 ± 11.5). In the study by Kramer et al. (1989), the main goal was to understand the relative impact of both age and alcohol abuse and, as such, they used two separate experimental and control groups. Young and old alcoholics presented a mean age of 37.2 (SD = 6.7) and 59.2 (SD = 3.8) respectively. Finally, one study did not present the necessary information to establish the participants mean age (Reich et al., 2004).

All four studies focusing on ecstasy/polydrug users (Fox et al., 2001; Schilt et al., 2008; Gallagher et al., 2012; Fisk et al., 2014) and two of the three studies with cannabis users (Kloft et al., 2019; Cuttler et al., 2021) presented a mean age between 20 and 30 years. One of the studies with ecstasy/polydrug users (Fox et al., 2001) separated the participants into those with a short and long-term history of use, presenting respective mean ages of 26.4 (SD = 5.9) and 30.7 (SD = 4.1). The remaining study with cannabis users (Riba et al., 2015) did not present the participants mean age.

Gender distribution throughout the studies varied between 23% to 100% male representation. Studies with individuals with a history of alcohol abuse or with an alcohol-use disorder tended to show disproportionately male samples. From the 10 studies, four had samples with over 70% male participants (Gudjonsson et al., 2000, 2004; Rocha and Albuquerque, 2003; Thoma et al., 2008). Three studies had samples where the male participants ranged between 40 and 60% (Maurage et al., 2011; Klein et al., 2013; Brion et al., 2017), and one (Reich et al., 2004) had a sample where only 23% of the participants were male. Studies focusing on ecstasy/polydrug users presented more evenly distributed samples regarding gender, with males representing between 50 and 60% of participants (Fox et al., 2001; Schilt et al., 2008; Fisk et al., 2014), even though in one of the studies (Fisk et al., 2014), the non-users group was mostly female (36% male). From the two studies on cannabis that provided data on gender distribution, one (Cuttler et al., 2021) presented between 50% and 70% of male representation across groups (average 56.3%) and the other (Kloft et al., 2019) over 80% on all but the control group (38%). Finally, the study with methadone maintenance patients (Henry et al., 2012) presented 43% and 37% of male representation for those with and without current cocaine dependence respectively.

Four studies did not present data on gender distribution (Kramer et al., 1989; Welch et al., 1997; Gallagher et al., 2012; Riba et al., 2015).

### 3.6. Substance type

Of the 18 included studies, 10 (55.55%) were conducted with individuals with a history of alcohol abuse or with an alcohol-use disorder (Kramer et al., 1989; Welch et al., 1997; Gudjonsson et al., 2000, 2004; Rocha and Albuquerque, 2003; Reich et al., 2004; Thoma et al., 2008; Maurage et al., 2011; Klein et al., 2013; Brion et al., 2017), four (22.22%) focused on ecstasy polydrug users (Fox et al., 2001; Schilt et al., 2008; Gallagher et al., 2012; Fisk et al., 2014) with one of these (Schilt et al., 2008) also considering the separate impact of other illicit substances, and three (16.67%) evaluated the impact of cannabis (Riba et al., 2015; Kloft et al., 2019; Cuttler et al., 2021). One study (5.56%) was done with methadone maintenance patients with current cocaine dependence (Henry et al., 2012).

### 3.7. Study characteristics

The characteristics of the studies [design, comparison group(s), type(s) of false memory, and false memory measures] are provided in Table 2.

**Table 2:** Study characteristics.

**References**	**Study design**	**Comparison group**	**Type(s) of false memory**	**False memory measure**
Kramer et al. (1989)	Quasi-experimental	Individuals without history of substance abuse	False recall & False recognition	California Verbal Learning Test (CVLT)
Welch et al. (1997)	Quasi-experimental	Individuals with temporal lobe epilepsy. Individuals with Parkinson's disease. Individuals with exposure to neurotoxins	Provoked confabulation	Visual Reproduction subtest of WMS-R (Card D)
Gudjonsson et al. (2000)	Experimental	Individuals with alcohol use disorder but evaluated 6 days after admission into the hospital (vs. 3 days)	Provoked confabulation	Confabulation subscale of Gudjonsson Suggestibility Scale
Fox et al. (2001)	Quasi-experimental	Individuals with a history of polydrug use	False recall	Auditory Verbal Learning Task (Immediate and Delayed recall)
Rocha and Albuquerque (2003)	Quasi-experimental	Individuals without history of substance abuse	False recall & False recognition	DRM paradigm
Gudjonsson et al. (2004)	Observational	NP	Provoked confabulation	Confabulation subscale of Gudjonsson Suggestibility Scale
Reich et al. (2004)	Quasi-experimental	Non-drinkers and Light-drinkers	False recall & False recognition	Adapted DRM paradigm (alcohol expectancy words)
Schilt et al. (2008)	Observational	NP	False recall & False recognition	Dutch version of the Rey Auditory Verbal Learning Test (RAVLT)
Thoma et al. (2008)	Quasi-experimental	Individuals without history of substance abuse	False recognition	List discrimination task
Maurage et al. (2011)	Quasi-experimental	Individuals without history of substance abuse	False recognition	“Confabulation task” (based on a continuous recognition paradigm)
Gallagher et al. (2012)	Quasi-experimental	Individuals without history of substance abuse (non-users)	False recognition	Word pair learning task
Henry et al. (2012)	Experimental	Methadone maintenance patients. without current cocaine dependence	False recognition	Recognition memory test
Klein et al. (2013)	Quasi-experimental	NP	False recognition	Recognition memory test
Fisk et al. (2014)	Quasi-experimental	Non-ecstasy using individuals	False recognition	Source memory task
Riba et al. (2015)	Quasi-experimental	Cannabis-naïve (< 50 lifetime occasions of cannabis use) healthy individuals	False recognition	DRM paradigm
Brion et al. (2017)	Quasi-experimental	Patients with Korsakoff's syndrome. Individuals without history of alcohol abuse	False recognition	Continuous recognition task
Kloft et al. (2019)	Quasi-experimental	Individuals with low past exposure to cannabis (< 10 lifetime occasions of cannabis use) Cannabis-intoxicated users	False recognition	DRM paradigm (word list administered as auditory stimuli)
Cuttler et al. (2021)	Quasi-experimental	Sober cannabis users	False recall & False recognition	DRM paradigm

NP, not provided.

### 3.8. Study design

From the 18 included studies, 14 (77.78%) have a quasi-experimental design (Kramer et al., 1989; Welch et al., 1997; Fox et al., 2001; Rocha and Albuquerque, 2003; Reich et al., 2004; Thoma et al., 2008; Maurage et al., 2011; Gallagher et al., 2012; Klein et al., 2013; Fisk et al., 2014; Riba et al., 2015; Brion et al., 2017; Kloft et al., 2019; Cuttler et al., 2021), two (11.11%) have an experimental design (Gudjonsson et al., 2000; Henry et al., 2012), and two (11.11%) have an observational design (Gudjonsson et al., 2004; Schilt et al., 2008).

### 3.9. Comparison group

Of the 18 studies, 14 (77.78%) included some type of comparison group. Eight studies (44.44%) used healthy individuals/ individuals without a history of substance abuse as the comparison group (Kramer et al., 1989; Rocha and Albuquerque, 2003; Reich et al., 2004; Thoma et al., 2008; Maurage et al., 2011; Gallagher et al., 2012; Riba et al., 2015; Kloft et al., 2019), one study (5.56%) used individuals with some type of neurological condition (Welch et al., 1997), and one study (5.56%) used both (Brion et al., 2017).

Six studies (33.33%) used individuals with some kind of substance use history as comparisons. Gudjonsson et al. (2000), compared two groups of individuals with alcohol-use disorder evaluated at different moments after admission into treatment. Fox et al. (2001), and Fisk et al. (2014), compared ecstasy users with polydrug users/ecstasy non-users. Henry et al. (2012), compared methadone maintenance patients with and without current cocaine dependence. Kloft et al. (2019), besides using a healthy control group, also used cannabis-intoxicated users as comparison group. Finally, Cuttler et al. (2021), compared sober cannabis users with cannabis users under the influence of different types of cannabis flower (varying potency).

Three studies (16.67%) did not use any comparison group (Gudjonsson et al., 2004; Schilt et al., 2008; Klein et al., 2013).

### 3.10. Type of false memory/false memory measures

From the 18 included studies, 15 studies (83.33%) focused on false recognition and/or false recall. Among these, the most used false memory procedure/task was the DRM paradigm, which was utilized in five studies (27.77%; Rocha and Albuquerque, 2003; Reich et al., 2004; Riba et al., 2015; Kloft et al., 2019; Cuttler et al., 2021). Two studies (13.33%) used a continuous recognition paradigm/task (Maurage et al., 2011; Brion et al., 2017), two (13.33%) used a type of recognition memory test (Henry et al., 2012; Klein et al., 2013), and two others (13.33%) administered some version of Rey Auditory Verbal Learning Test (Fox et al., 2001; Schilt et al., 2008; RAVLT). The four remaining studies (26.67%) used different measures. Kramer et al. (1989) used the California Verbal Learning Test (CVLT), Thoma et al. (2008) used a list discrimination task, Fisk et al. (2014) applied a source memory task, and Gallagher et al. (2012) used a word pair learning task.

From the three studies that focus on provoked confabulation, two (66.67%) used the Confabulation Subscale of Gudjonsson Suggestibility Scale (Gudjonsson et al., 2000, 2004), and one (33,33%) the Visual Reproduction subtest of WMS-R (Card D; Welch et al., 1997).

In Table 3, a detailed description of the used measures is provided.

**Table 3:** False memory measures and outcomes.

**False memory measure**	**Description**	**Outcomes**	**Used in** ***(n*****)**	**References**
**False recall and false recognition**				
California Verbal Learning Test (CVLT)	Five learning trials of a 16-word target list (four words for each semantic category). After each trial, participants recall as many words as possible. An interference list is then presented for a learning trial, and after a short delay, participants recall the target list. Long delay free recall, cue recall and recognition are assessed after 20 min. Recognition task uses yes/no paradigm, 16 targets and 28 distractors. Distractors consist of interference words (both semantically related and semantically unrelated to targets), and novel words (prototypical of semantic categories, phonemically similar, neither semantically nor phonemically related to the targets).	Free immediate, short-delay and long-delay recall. Intrusions during recall (false recall) Corrects hits on recognition False positives on recognition (false recognition assessed through false positives regarding novel prototype words)	1	Kramer et al., 1989
Continuous recognition paradigm/task	Two blocks, being the first a recognition task composed of 6 trials, each with a sequence of 20 black-and-white drawings of animals or real objects (the same 8 targets on each sequence, but 12 different distractors). Each trial consists in a 700-ms fixation cross, followed by the drawing items. Participants have to decide if each drawing was presented previously in the current trial. Second block is presented 1 h after the first one and uses the same procedure. In this block, targets items are replaced so that eight distractors from the first block become target items (and target items from the first block become distractors). Instructions remain the same.	For each block: Reaction time, Number of hits, False alarms Temporal context confusion (TCC) - relative increase of false alarms in the second run as compared to the first (false recognition measure) TCC = (FP2/Hits2) - (FP1/Hits1)	2	Maurage et al., 2011; Brion et al., 2017
DRM Paradigm	A study phase, in which the participants either read or hear a variable number of word lists, and a testing phase in which the participants are asked to freely recall or/and recognize (from a presented list) the previously studied words. Each of the lists of words presented in the study phase is semantically related to a non-presented word (critical lure). In the recognition test, the critical lure (along with other related and unrelated words) is included in the presented list. Rocha and Albuquerque (2003) used eight lists with seven Portuguese words each and assessed both free recall (2,5 min per list) and recognition (3 min after the recall of the last list). In the recognition condition, confidence level was also assessed. Reich et al. (2004) used six lists, five neutral lists to establish baseline and one target list with either alcohol expectancy words (experimental group) or nonalcohol expectancy adjectives (control group). The target list was always presented fourth in the sequence. Target lists were significantly different from the word lists traditionally used in the paradigm, namely because they were composed by adjectives (instead of nouns) and had almost no semantic association with the target words. Riba et al. (2015) used 20 lists of four words. Fifteen lists were composed of four semantically related Spanish words, and the remaining lists were composed of three semantically related words and one catch word (to control for the subjects' attention). The authors only considered the recognition condition. Kloft et al. (2019) used 10 lists of 10 words each. The authors only assessed recognition and not free recall. Finally, Cuttler et al. (2021) used six lists of 12 words each. They assessed free recall immediately after the participants heard each list. Recognition was assessed after a 10-min retention interval.	Hit rate False alarm rate for critical lures False alarm rate for related words False alarm rate for unrelated words Net accuracy	5	Rocha and Albuquerque, 2003; Reich et al., 2004; Riba et al., 2015; Kloft et al., 2019; Cuttler et al., 2021
List discrimination task	Six study-test blocks, each with two study lists (16 items per list) and two test lists (24 items per list). Test lists include eight words each study list, and eight novel words presented in random order. Participants are instructed to make confidence judgements (six-point scale from “certain yes” to “certain no”) relating to the list membership of each item. The first and second study lists each serve as target lists (and are tested first) in half the test blocks.	Hit rate False alarm rate to items from non-target list. False alarm rate to novel items Old/new recognition score and discrimination score	1	Thoma et al., 2008
Recognition memory test	In Klein et al. (2013), they presented words and asked the participants if they had seen them in an earlier attentional task. Sixteen (eight alcohol and eight gemstone) words had been previously presented and 16 were new (four related to alcohol and four related to gemstones). The remaining distractors were unrelated to each category or to each other. In Henry et al. (2012), the recognition memory test was not described.	For each word type: Hit rate, False alarm rate d prime (discrimination score) beta (bias measure)	2	Henry et al., 2012; Klein et al., 2013
Rey Auditory Verbal Learning Test (RAVLT)	Five learning trials in which the participants are presented with a list of 15 words at the rate of one word per second each. Each trial is followed by immediate recall. An interference list of 15 words is then presented, after which recall of the target list is tested. In Fox et al. (2001), after the fifth trial, a new 15-word list was presented followed by immediate recall. Afterwards, the participants were asked to once again recall the first list. Delayed recall was assessed after 30 minutes (0.5 h) and recognition was not assessed. In Schilt et al. (2008), a second list was not presented. Delayed recall and recognition were assessed after 20 min.	Memory score (total correct words out of 15) Total incorrect words unrelated to the stimulus list Total incorrect words semantically or phonetically related to the stimulus list Intrusion errors from one list into the other (in Fox et al., 2001).	2	Fox et al., 2001; Schilt et al., 2008
Source memory task	In the study phase, two lists of 32 words are presented, with each word being displayed on the screen for 4s. Half the words in each list are presented in the top section of the computer screen, and the other half on the bottom section. Similarly, half the words are presented with upper case and half with lower case. In the recognition phase, all 64 words from the studied lists are presented along with 64 new words. In Fisk et al. (2014) both recognition and source memory were assessed. Participants were asked to decide if each word had been presented previously, as well as indicate its position, format and original list.	Number of hits False positive responses Estimate of sensitivity Percentage of correct source memory judgements (list, position, format)	1	Fisk et al., 2014
Word pair learning task	In the encoding phase, 80 word pairs (two common concrete nouns) are presented in a randomized order, each displayed for 4 s in the computer screen (500-ms gap). The recognition phase immediately follows the encoding phase, and consists of the initial 80 words pairs and an additional 60 new ones. Words pairs can be old word pairs (previously presented), a new conjunction (with previously presented words that had not belong in the same word pair), a new item (one previously presented word and one new word), and new word pair (both words are new). Each of these four word pair types appears 15 times during recognition. Participants are asked to decide if a word pair is old (previously presented as a word pair) or new (conjunction, item, or word pair). In Gallagher et al. (2012) recognition was assessed under single attention and divided attention (with a digit-monitoring task in the encoding phase) conditions.	Number of hits False alarms Mean number of old responses (for each of the word pair types)	1	Gallagher et al., 2012
**Provoked confabulation**				
Confabulation score of Gudjonsson Suggestibility Scale	Evaluates confabulation in memory recall and considers a distinction between “distortions” (minor alteration in memory) and “fabrications” (significant new information being added).	Distortion score. Fabrication score. Confabulation score	2	Gudjonsson et al., 2000, 2004
Visual Reproduction subtest of WMS-R (Card D)	In Welch et al. (1997) the visual reproduction subtest of the WMS-R was administered in the standard format. Productions of the Card D created by the participants were later examined for changes that might make the figure resemble a drinking vessel (e.g., wine glass).	Modifications in the Card D productions: 90-degree rotation Embellishment into glass-like figure. Comments about having been shown a drinking vessel for any type of alcohol	1	Welch et al., 1997

### 3.11. False memory outcomes

From the 15 studies focused on false recognition/false recall, only five (33.3%) considered false recognition or false recall of critical lures (Rocha and Albuquerque, 2003; Reich et al., 2004; Riba et al., 2015; Kloft et al., 2019; Cuttler et al., 2021). Of these, three (60%) considered both false recognition and false recall of critical lures (Rocha and Albuquerque, 2003; Reich et al., 2004; Cuttler et al., 2021), and two (40%) considered only false recognition of critical lures (Riba et al., 2015; Kloft et al., 2019). Another study took into account both false recognition and false recall of novel prototype words (Kramer et al., 1989).

Five studies (33.3%) considered false recognition/false recall of related items (Kramer et al., 1989; Fox et al., 2001; Rocha and Albuquerque, 2003; Reich et al., 2004; Cuttler et al., 2021). Three inspected both false recognition and false recall of related items (Kramer et al., 1989; Rocha and Albuquerque, 2003; Cuttler et al., 2021), one considered only false recognition of related items (Reich et al., 2004), and another only false recall of related items (Fox et al., 2001).

Six studies (40%) dealt with false recognition/false recall of unrelated items (Kramer et al., 1989; Fox et al., 2001; Rocha and Albuquerque, 2003; Reich et al., 2004; Kloft et al., 2019; Cuttler et al., 2021). Three considered both false recognition and false recall of unrelated items (Kramer et al., 1989; Rocha and Albuquerque, 2003; Cuttler et al., 2021), two analyzed only false recognition of unrelated items (Reich et al., 2004; Kloft et al., 2019), and one considered only false recall of unrelated items (Fox et al., 2001).

Seven studies (46.7%) considered a measure of false alarm/false positive rate without specifying the type of items included (e.g., related, unrelated; Schilt et al., 2008; Maurage et al., 2011; Henry et al., 2012; Klein et al., 2013; Fisk et al., 2014; Riba et al., 2015; Brion et al., 2017). Finally, four studies (26.7%) looked at other types of intrusion errors such as novel items and non-target list items (Thoma et al., 2008), temporal context confusion (TCC; Maurage et al., 2011; Brion et al., 2017), and old, new conjunction, new item, and new word pairs (Gallagher et al., 2012).

From the three studies that focused on provoked confabulation, two took into account distortion, fabrication, and confabulation scores (Gudjonsson et al., 2000, 2004), and one considered modifications to the WMS-R Card D such as a 90-degree rotation and embellishment into a glass-like figure (Welch et al., 1997). A detailed description of the outcomes used by each study can be found in Table 3.

### 3.12. Key findings

Table 4 reports the main results of interest for the 18 studies included in the present review. From the 10 studies focusing on individuals with a history of alcohol abuse (Kramer et al., 1989; Welch et al., 1997; Gudjonsson et al., 2000, 2004; Rocha and Albuquerque, 2003; Reich et al., 2004; Thoma et al., 2008; Maurage et al., 2011; Klein et al., 2013; Brion et al., 2017), two (20%; Rocha and Albuquerque, 2003; Reich et al., 2004) used the DRM paradigm for obtaining a measure of false recognition/false recall, five (50%; Kramer et al., 1989; Thoma et al., 2008; Maurage et al., 2011; Klein et al., 2013; Brion et al., 2017) employed other procedures which considered different types of intrusion errors, and three (30%; Welch et al., 1997; Gudjonsson et al., 2000, 2004) focused on provoked confabulations. Neither of the studies that used the DRM paradigm (100%)–one in alcoholic patients (Rocha and Albuquerque, 2003) and the other in light and heavy drinkers (Reich et al., 2004)–found significant differences regarding the false recognition/false recall of critical lures concerning the samples studied in each of these studies. However, Rocha and Albuquerque (2003) found that alcoholic patients showed a significantly higher rate of other intrusion errors in the free recall task than controls. Interestingly, Reich et al. (2004), reported that heavy drinkers registered a significant increase in false recognition rates (for target alcohol expectancy words) when in an alcohol-related context (bar). This was not true for participants with a lighter drinking pattern. Regarding the other studies measuring false recognition/false recall, 3 (75%; Kramer et al., 1989; Thoma et al., 2008; Maurage et al., 2011) of the four studies (Kramer et al., 1989; Thoma et al., 2008; Maurage et al., 2011; Brion et al., 2017) with a control group found significant differences indicating that individuals with a history of alcohol abuse have a higher susceptibility to this phenomenon. Kramer et al. (1989), using the CVLT, found that alcoholics presented significantly more intrusions than controls across all false positive types, including novel prototype words. Similarly, Thoma et al. (2008), showed that alcohol-dependent participants presented significantly higher false alarm rates (both to non-target list items and to novel items) in a list discrimination task. Maurage et al. (2011), reported that alcoholics had a significantly higher temporal context confusion (TCC) index in a continuous recognition paradigm (“confabulation task”). However, Brion et al. (2017), who also employed a continuous recognition task, did not find similar results. According to their study, only patients with Korsakoff Syndrome showed a higher rate of temporal context confusions when compared to both alcoholic patients (without that syndrome) and controls. Lastly, Klein et al. (2013), who did not use a comparison group, found that patients receiving treatment for alcohol dependence presented significantly higher hit and false alarm rates for alcohol-related words when compared with neutral words. The only study (100%) focused on provoked confabulations that used a comparison group (Welch et al., 1997) did not find the presence of confabulation in our target population. Welch et al. (1997), used the Card D of the visual reproduction subtest of the WMS-R and found that only alcoholics with brain damage presented spontaneously produced alterations that resembled “drinking vessels”. The remaining studies (Gudjonsson et al., 2000, 2004) sought not to compare individuals with a history of alcohol abuse with other populations, but to investigate the impact of withdrawal on suggestibility. Gudjonsson et al. (2000), did not find any significant differences on the suggestibility scores (including the confabulation subscale) between patients assessed at the beginning of their hospital admission or after at least 6 days of hospitalization. However, in the second study (Gudjonsson et al., 2004) it was observed that the severity of withdrawal symptoms was significantly associated to confabulation and other suggestibility scores. None of the studies focusing on ecstasy/polydrug use utilized the DRM paradigm for measuring false recognition/false recall. From the four studies that looked into this population (Fox et al., 2001; Schilt et al., 2008; Gallagher et al., 2012; Fisk et al., 2014), 3 (75%) it was found that ecstasy users showed significantly higher rates of intrusions than non-ecstasy users (Fox et al., 2001; Schilt et al., 2008; Gallagher et al., 2012). Fox et al. (2001) and Schilt et al. (2008), both using a version of Rey Auditory Verbal Learning Test (RAVLT), reported that ecstasy users had significantly more intrusion errors than short-term users and controls and non-ecstasy users respectively. Gallagher et al. (2012), while studying the associate learning processes of ecstasy/polydrug users through a word pair learning task, found that ecstasy users showed significantly higher false alarm rates both in the single attention condition (to conjunction and new word pairs) and in the divided attention condition (to conjunction, new and item word pairs). Finally, Fisk et al. (2014), interested in understanding the impact of ecstasy/polydrug use on source memory processes, observed that ecstasy/polydrug users did not differ significantly from non-users regarding hits, false positives rates and sensitivity, even though they were worst in case source memory judgement. All the studies investigating false memories with cannabis users (Riba et al., 2015; Kloft et al., 2019; Cuttler et al., 2021) included in our review utilized the DRM paradigm. Of the two studies with a healthy control group, one (50%; Kloft et al., 2019) did not find a significant difference in the false recognition rates to critical lures between cannabis users and controls but found a significantly higher false recognition rate for unrelated words. This study administered the word list as auditory stimuli. The other (Riba et al., 2015) reported different results, indicating a higher susceptibility to false memories for cannabis users, with significantly higher rates of false recognition (including to critical lures) and lower rates of false memory rejection. Moreover, this higher susceptibility to false recognition appeared associated with a hypoactivation of several spatially distributed brain regions involved in semantic and episodic retrieval. Lastly, Cuttler et al. (2021), who compared different high-potency cannabis flowers and concentrates (but did not use a healthy control), indicated that cannabis intoxication impaired source memory, but did not significantly increase false recognition rates for critical lures. The only study (Henry et al., 2012) that sought to compare the cognitive performance of methadone maintenance patients with and without cocaine dependence, found that patients with cocaine dependence showed a significantly higher false alarm rate in a recognition memory test.

**Table 4:** Key findings.

**References**	**Study objective(s)**	**Key findings**
**False recall and false recognition**		
Kramer et al. (1989)	Test the premature aging hypothesis of alcoholism. More specifically if alcoholism leads to premature aging of memory functioning.	The results did not support either variant of the premature aging hypothesis, indicating that the effects of aging and alcohol abuse on verbal learning represent different phenomenon. Regarding false recall, alcoholics presented more intrusions (across false positive types, including novel prototype words) than controls.
Fox et al. (2001)	Investigate how the deficits on verbal working memory and memory span seen in drug-free ecstasy polydrug users, affect the learning of verbal material. Examine potential differences between short and long-term Ecstasy polydrug users and polydrug users who never consumed Ecstasy (or used it on two or less occasions).	Ecstasy user groups recalled significantly fewer words both on the initial trials (1, 2, 3) and on the delayed recall phase (Trial 7). Long-term ecstasy users (vs. short-term ecstasy users and controls) made more combined (intrusion and association) errors on the immediate recall component of AVLT and list B.
Rocha and Albuquerque (2003)	Analyze memory deficits and the occurrence of memory illusions (false recall and false recognition) in alcoholics using the DRM paradigm.	The experimental group's average of recalled words was significantly lower than the control group, which supports a higher recall capacity by the control group. There were no significant differences between the groups regarding false recall or false recognition of critical lures, but there were significant differences in the free recall of other intrusions (with the experimental group showing a higher percentage).
Reich et al. (2004)	Test two assumptions of the alcohol expectancy theory, which say that memories relating to alcohol effects are stored as templates of information and that these are automatically activated when in alcohol-related contexts. The hypotheses were that: (1) participants would present more false memories for alcohol expectancy words in an alcohol-related context; (2) participants with a history of heavy drinking would present more false memories in an alcohol-related context (vs neutral context).	The results were consistent with both studied components from the alcohol expectancy theory. All participants (non-drinkers, light-drinkers, and heavy-drinkers) showed similar results in standard DRM lists. Participants with a heavy drinking pattern presented significantly more false memories in an alcohol-related context (bar) than in a neutral context. This difference was not significant for participants with lower drinking patterns.
Schilt et al. (2008)	Investigate the sustained effects of ecstasy on cognitive functioning (independent of other substances) using a neuropsychological examination.	Ecstasy independently accounted for significant verbal memory impairments. Ecstasy users (vs. non-users) recalled fewer words and presented more intrusion errors.
Thoma et al. (2008)	Study the effects of chronic alcohol consumption on recollection and familiarity using a verbal list discriminating task and analyses based on the dual process signal detection model (DPSD) and the process dissociation procedure (PDP).	Alcohol-dependent participants showed significant recollection impairment in both DPSD and PDP analyses, but only showed familiarity impairment according to analyses based on the PDP. Alcohol-dependent participants presented significantly higher false alarms rates (both to non-target list and to novel items) than healthy comparators.
Maurage et al. (2011)	Explore the association between olfactory and executive functions in alcoholic individuals and investigate the usefulness of olfaction as a cognitive market of psychiatric states.	High-level olfactory functions and executive functions implicating the orbitofrontal cortex were positively correlated in both alcoholics and controls. Alcoholics showed a significant impairment for high-level olfactory processing and a significantly higher temporal context confusion (TCC) index.
Gallagher et al. (2012)	Study the associate learning processes of ecstasy/polydrug users using a word pair learning task.	Ecstasy users showed higher false alarm rates (vs. non-users) to conjunction and new word pairs in the single attention condition and to conjunction, new and item word pairs in the divided attention condition.
Henry et al. (2012)	Compare the cognitive performance of methadone maintenance patients with and without cocaine dependence using a standard battery of tests.	Patients with cocaine dependence (vs without cocaine dependence) only showed significantly more impairment on some psychomotor performance/attention and episodic memory measures. Patients with cocaine dependence had a significantly higher false alarm rate but similar hit rate to patients without cocaine dependence.
Klein et al. (2013)	Investigate the presence of attention and recognition memory biases for alcohol-related stimuli in patients admitted into a residential treatment for alcohol dependence.	Patients presented a significant cognitive processing bias for alcohol-related stimuli. Both hit and false alarm rates were higher for alcohol-related words than for neutral words.
Fisk et al. (2014)	Examine the impact of ecstasy/polydrug use on source memory processes by comparing ecstasy/polydrug users with non-users.	The performance of ecstasy/polydrug users and non-using controls did not differ significantly regarding hits, false positives, and sensitivity. Ecstasy/polydrug users were significantly worse than non-using controls in letter case judgement.
Riba et al. (2015)	Investigate the impact of chronic cannabis use on the ability to distinguish between veridical and illusory memories, that is, on the susceptibility to false memories.	Cannabis users presented a significantly higher susceptibility to false memories, with higher rates of false recognition and lower rates of false memory rejection. This deficit appeared associated with a hypoactivation of a number of distributed brain regions involved in semantic and episodic retrieval.
Brion et al. (2017)	Explore the source memory deficits presented by patients with Korsakoff Syndrome using a “continuous recognition task”.	Patients with Korsakoff Syndrome showed a higher rate of temporal context confusions, registering source memory impairments. Patients with alcohol dependence (but without Korsakoff Syndrome) did not show similar source memory deficits. Patients with Korsakoff Syndrome presented significantly more false detections than patients with alcohol dependence or healthy comparators.
Kloft et al. (2019)	Investigate if cannabis use leads to an increase of the susceptibility to false memory formation.	Cannabis users (vs. non-users) did not show a significantly higher false memory rate for critical lures. However, both intoxicated and sober cannabis users presented a significantly higher false recognition rate for unrelated items.
Cuttler et al. (2021)	Examine acute effects of both high-potency cannabis flower and concentrates on memory and decision-making.	Cannabis significantly impaired free recall and increased false memory rates for related and unrelated words. Cannabis did not significantly increase false recognition rates for critical lures. Cannabis showed a detrimental effect on source memory.
**Provoked confabulation**		
Welch et al. (1997)	Evaluate a potential specific visual confabulation in alcoholic patients by conducting a retrospective search for “wineglass” rotations after the administration of the Card D of the visual reproduction subtest of the WMS-R.	Twenty percent of the group of alcoholics with brain damage (6 patients out of 30) presented spontaneously produced alterations to the Card D resembling the bowl and stem of a drinking vessel. None of the alcoholic controls or the patients in the other groups made this alteration.
Gudjonsson et al. (2000)	Investigate the impact of alcohol withdrawal on the accuracy of information obtained in an interview and on the ability to cope with interrogative pressure. This was done by comparing the levels of suggestibility and compliance of patients who were tested at the beginning of their hospital admission, and patients who had been hospitalized for at least 6 days.	Suggestibility scores did not differ significantly between the two groups, despite a significant difference regarding memory, overall cognitive functioning, and anxiety levels.
Gudjonsson et al. (2004)	Investigate the relationship between suggestibility and alcohol withdrawal in male and female alcoholics.	The severity of withdrawal symptoms was significantly associated to memory, confabulation, suggestibility, and compliance scores. This relationship differed according to sex. For males, severity of withdrawal symptoms was negatively associated with memory performance. For females, severity of withdrawal symptoms was negatively associated with distortions and positively associated with fabrications.

### 3.13. Risk of bias

To assess the risk of bias of the Randomized Controlled Trials, we used the Cochrane Risk of Bias Tool (Higgins et al., 2011). The quasi-experimental studies were evaluated for risk of bias using the Joanna Briggs Institute JBI Critical Appraisal Checklist for Quasi-Experimental Studies (non-randomized experimental studies). In turn, the JBI Critical Appraisal Checklist for Analytical Cross Sectional Studies was used for the assessment of the observational studies.

In the two randomized controlled trials (Gudjonsson et al., 2000; Henry et al., 2012), the methodological issues are not detailed or are incomplete, therefore the risk of bias assessment was limited (information provided in Table 5).

**Table 5:** Risk-of-bias assessment of the randomized controlled trials.

**References**	**Random sequence generation (selection bias)**	**Allocation concealment (selection bias)**	**Blinding of participants and personnel (performance bias)**	**Blinding of outcome assessment (detection bias)**	**Incomplete outcome data (attrition bias)**	**Selective reporting (reporting bias)**	**Other bias**
Gudjonsson et al. (2000)	+	+	?	?	?	?	?
Henry et al. (2012)	?	?	-	?	?	?	?

- Indicates low risk of bias; + indicates high risk of bias; ? indicates unclear risk of bias.

Regarding quasi-experimental studies (Kramer et al., 1989; Welch et al., 1997; Fox et al., 2001; Rocha and Albuquerque, 2003; Reich et al., 2004; Thoma et al., 2008; Maurage et al., 2011; Gallagher et al., 2012; Klein et al., 2013; Fisk et al., 2014; Riba et al., 2015; Brion et al., 2017; Kloft et al., 2019; Cuttler et al., 2021), it was found that most studies did not include a follow-up, for that reason this factor was not applicable (see Table 6).

**Table 6:** Risk-of-bias assessment of quasi-experimental studies (Joanna Briggs Institute tool).

**References**	**Q1**	**Q2**	**Q3**	**Q4**	**Q5**	**Q6**	**Q7**	**Q8**	**Q9**
Kramer et al. (1989)	Y	Y	NA	Y	NA	NA	Y	Y	Y
Welch et al. (1997)	Y	Y	?	Y	N	NA	Y	Y	Y
Fox et al. (2001)	Y	Y	NA	Y	N	NA	Y	Y	Y
Rocha and Albuquerque (2003)	Y	Y	NA	Y	N	NA	Y	?	?
Reich et al. (2004)	Y	Y	Y	N	N	NA	Y	Y	Y
Thoma et al. (2008)	Y	Y	Y	Y	N	NA	Y	Y	Y
Maurage et al. (2011)	Y	Y	Y	Y	N	NA	Y	Y	Y
Gallagher et al. (2012)	Y	Y	NA	Y	N	NA	Y	Y	Y
Klein et al. (2013)	Y	Y	Y	N	N	NA	Y	Y	Y
Fisk et al. (2014)	Y	Y	NA	Y	N	NA	Y	Y	Y
Riba et al. (2015)	Y	Y	N	Y	N	NA	Y	Y	Y
Brion et al. (2017)	Y	Y	N	Y	N	NA	Y	Y	Y
Kloft et al. (2019)	Y	Y	N	Y	N	N	Y	Y	Y
Cuttler et al. (2021)	Y	Y	N	Y	Y	N	Y	Y	Y

Y indicates yes, N indicates no, ? indicates unclear, NA indicates not applicable.

Q1–Is it clear in the study what is the “cause” and what is the “effect” (i.e. there is no confusion about which variable comes first)?

Q2–Were the participants included in any comparisons similar?

Q3–Were the participants included in any comparisons receiving similar treatment/care, other than the exposure or intervention of interest?

Q4–Was there a control group?

Q5–Were there multiple measurements of the outcome both pre and post the intervention/exposure?

Q6–Was follow up complete and if not, were differences between groups in terms of their follow up adequately described and analyzed?

Q7–Were the outcomes of participants included in any comparisons measured in the same way?

Q8–Were outcomes measured in a reliable way?

Q9–Was appropriate statistical analysis used?

Only two studies (Kloft et al., 2019; Cuttler et al., 2021) showed the existence of follow-up, however, the differences between the groups were not adequately described. It was also found that most of the studies included in the present review did not measure the outcomes before and after the intervention. This presupposition was only verified in the study (Cuttler et al., 2021).

In the observational studies (Gudjonsson et al., 2004; Schilt et al., 2008), the most common sources of bias relate to a poor identification of confounding factors and/or a lack of appropriate strategies to mitigating them (see Table 7).

**Table 7:** Risk-of-bias assessment of observational studies (Joanna Briggs Institute tool).

**References**	**Q1**	**Q2**	**Q3**	**Q4**	**Q5**	**Q6**	**Q7**	**Q8**
Gudjonsson et al. (2004)	N	N	?	Y	?	?	Y	Y
Schilt et al. (2008)	Y	Y	Y	Y	?	?	Y	Y

Y indicates yes, N indicates no, ? indicates unclear.

Q1–Were the criteria for inclusion in the sample clearly defined?

Q2–Were the study subjects and the setting described in detail?

Q3–Was the exposure measured in a valid and reliable way?

Q4–Were objective, standard criteria used for measurement of the condition?

Q5–Were confounding factors identified?

Q6–Were strategies to deal with confounding factors stated?

Q7–Were the outcomes measured in a valid and reliable way?

Q8–Was appropriate statistical analysis used?

The risk of bias assessment identified was discussed between two reviewers (TC; JL). In situations where there were discrepancies and no consensus was reached, the intervention of a third reviewer (TA) was considered.

### 3.14. Meta-analysis

From the 18 studies included in systematic review, only five (27.8%) were eligible to be included in the meta-analysis since the articles of the remaining studies did not present the necessary data. The authors were contacted for the missing data but only two responded. One study (Schilt et al., 2008) was excluded because it used different comparator groups (ecstasy-users vs. non-ecstasy users) and there were no other studies to allow for a subgroup analysis.

From the five studies that were included in the meta-analysis, two (40%) presented results on the false recognition of critical lures (Riba et al., 2015; Kloft et al., 2019), two (40%) presented results on the false recognition of unrelated items (Thoma et al., 2008; Kloft et al., 2019), and three (60%) presented results on other intrusion error outcomes such as TCC (Maurage et al., 2011; Brion et al., 2017), and false alarm for items of a non-target list (Thoma et al., 2008). Separate analyses were conducted for each of these outcomes.

With regard to false recognition of critical lures, the analysis indicated that there were no significant differences between individuals with a history of substance abuse (in this case, cannabis) and individuals without a history of substance abuse (SMD = 0.26; Z = 0.56; *p* = 0.57). Heterogeneity between the studies was high, with χ^**2**^ = 4.79, *p* = 0.03, and I^2^ = 79%. Both of the studies included in this analysis used the DRM paradigm as a measure of false memory and, as such, there was no subgroup analysis.

Concerning false recognition of unrelated items, the analysis indicated that individuals with a history of substance abuse presented significantly higher rates than individuals without a history of substance abuse when measured both by the DRM paradigm (SMD = 0.59; Z = 2.94; *p* = 0.003) and by a list discrimination task (SMD = 1.10; Z = 3.18; *p* = 0.001). The test for subgroup differences did not find a statistically significant subgroup effect (*p* = 0.20).

Finally, the analysis looking into other type of intrusion errors found that individuals with a history of substance abuse presented significantly higher rates of false alarms to non-target list items in a list discrimination task (SMD = 0.94; Z = 2.78; *p* = 0.005) but not regarding TCC in a continuous recognition paradigm (SMD = 0.38; Z = 0,80; *p* = 0.42) when compared with individuals with healthy controls. Heterogeneity between the studies in the continuous recognition paradigm subgroup was high, with χ^**2**^ = 4,23, *p* = 0.04, and I^2^ = 76%. There was no statistically significant subgroup effect (*p* = 0.34).

**Figure 2:**
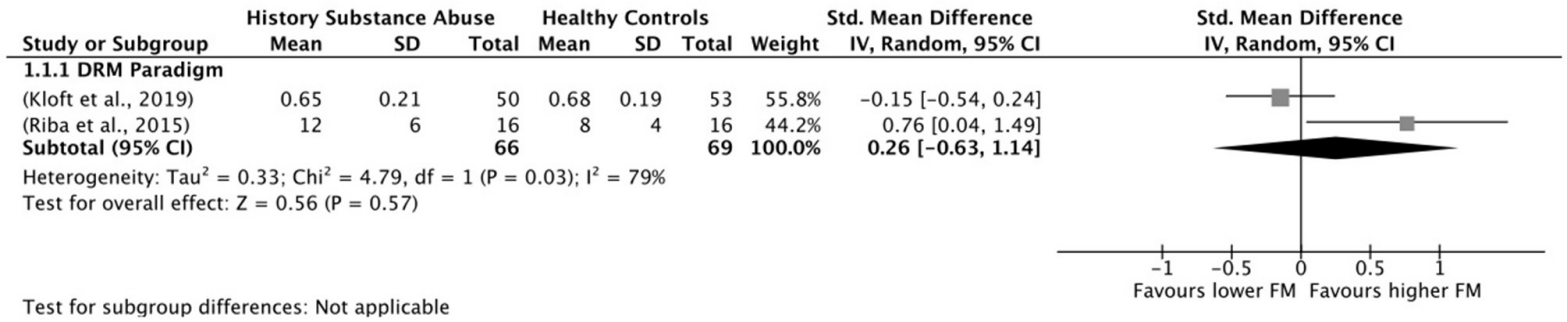
Forest plot of false recognition of critical lures. FM, false memories.

**Figure 3:**
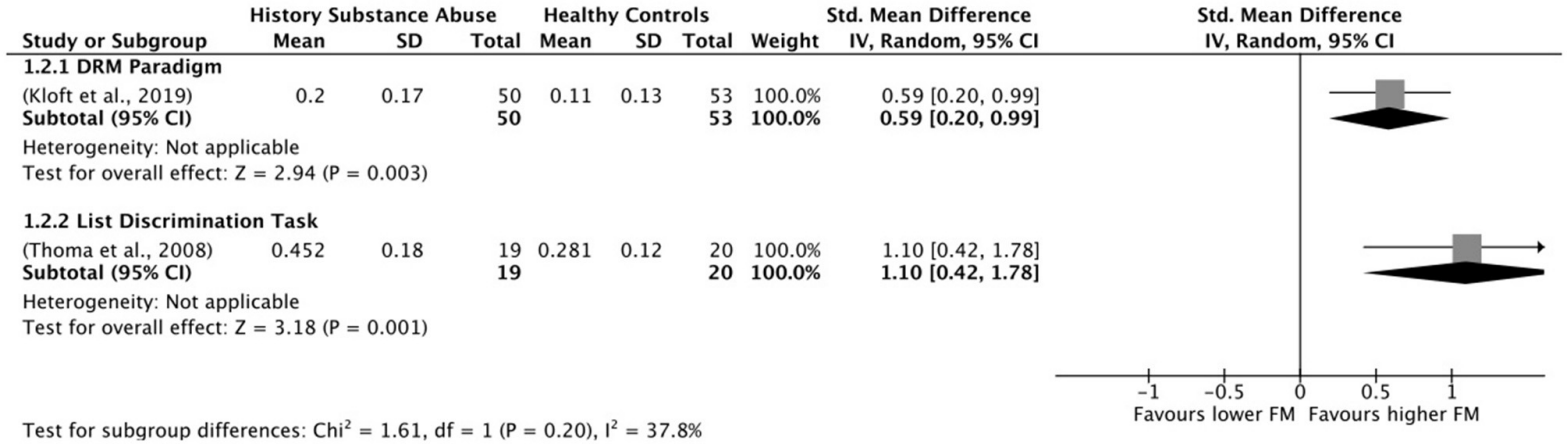
Forest plot of false recognition of unrelated items. FM, false memories.

**Figure 4:**
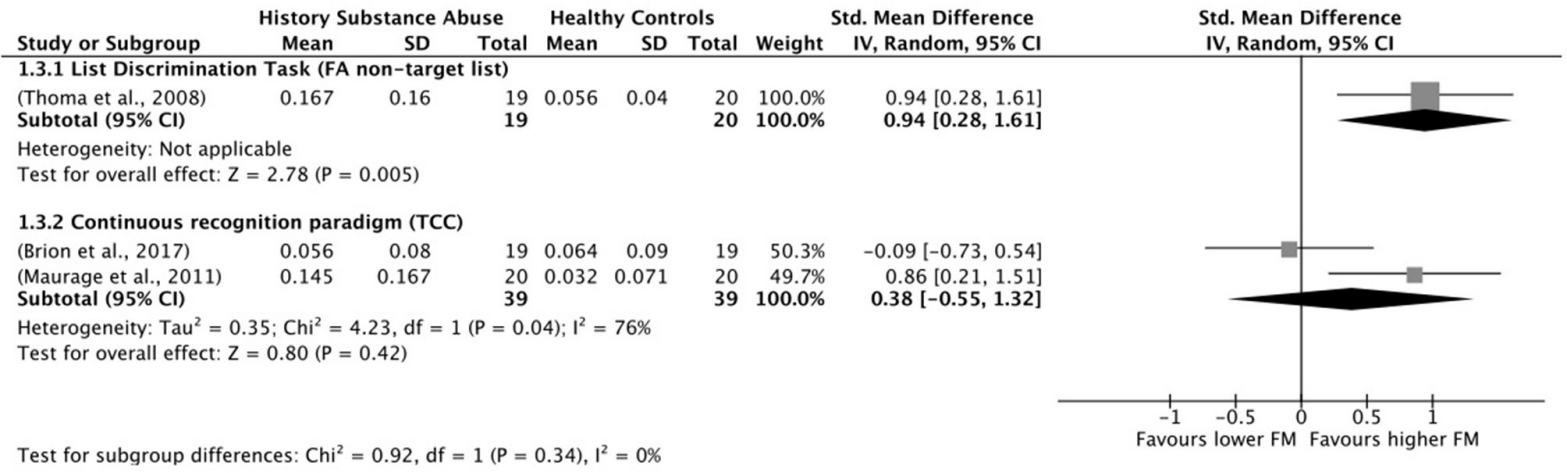
Forest plot of other intrusion errors. FM, false memories.

The graphic representation for each of the analyses can be found on Figures 2–4 respectively.

## 4. Discussion

The main goal of this systematic review was to synthesize the current scientific knowledge regarding the relationship between substance abuse and susceptibility to false memory formation. In view of the lack of clarity surrounding the conceptualization of the different types of false memories, and the resulting absence of consensus on how to measure them, this was not a straightforward endeavor. For the purpose of this review, we considered three main types of false memories: false recognition/false recall, provoked confabulation, and spontaneous confabulation. As expected, we only found studies focusing on the first two, since spontaneous confabulation is very difficult to measure. We also chose to include any study that presented a measure of false recognition/false recall of items other than critical lures (as measured by the DRM paradigm), with the goal of getting a broader understanding of the impact of substance abuse on false memory and hopefully contribute for the clarification of the associated concepts.

The results suggest that the mentioned clarification is crucial and that different false memory types or intrusion errors (i.e., false recognition/false recall of critical lures, false recognition of other related items, false recognition of unrelated items, provoked confabulations) should be considered independently not only when studying the potential impact of substance abuse but whenever false memory is a topic of interest. In this light, we will discuss the results according to our original research questions, along with the different measures considered in the included studies.

### 4.1. Individuals with a history of substance abuse vs. healthy individuals

Half of the studies included in the present review used a healthy comparison group (Kramer et al., 1989; Rocha and Albuquerque, 2003; Reich et al., 2004; Thoma et al., 2008; Maurage et al., 2011; Gallagher et al., 2012; Riba et al., 2015; Brion et al., 2017; Kloft et al., 2019), and all of these focused on false recognition/false recall.

The majority of the studies that considered false recognition and false recall of critical lures through the use of the DRM paradigm (Rocha and Albuquerque, 2003; Reich et al., 2004; Kloft et al., 2019) did not indicate an increased susceptibility in individuals with a history of substance abuse when compared to healthy controls. This was true for all studies with alcoholics (Rocha and Albuquerque, 2003) or heavy alcohol users (Reich et al., 2004) and for one study with cannabis-users (Kloft et al., 2019). Only one study (Riba et al., 2015), also looking into the potential impact of cannabis, found different results, with cannabis-users presenting significantly higher false recognition rates for critical lures. The meta-analysis focusing on this particular outcome did not find any significant differences between individuals with a history of substance abuse and healthy controls.

Considering the low number of studies included in the present review, it is difficult to understand if there are any variables that may have contributed for this difference in results. Nonetheless, looking into the studies by Riba et al. (2015) and Kloft et al. (2019), which both focus on cannabis, we can speculate that the duration and frequency of cannabis use may be an important variable for future studies to consider. While in the study by Kloft et al. (2019), the individuals in the experimental group were described as regular cannabis users, in Riba et al. (2015), they were described as heavy cannabis users (defined by daily use for the last 2 years, with an average of 21 years of use and an average of five joints per day).

When considering other intrusion errors, the results were reversed, with a majority of the studies (Kramer et al., 1989; Rocha and Albuquerque, 2003; Thoma et al., 2008; Maurage et al., 2011; Gallagher et al., 2012; Kloft et al., 2019) indicating higher susceptibility for individuals with a history of substance abuse when compared with healthy comparators. This was true for the false recognition/false recall of related (Kramer et al., 1989; Rocha and Albuquerque, 2003) or non-target list items (Thoma et al., 2008) and unrelated (Kramer et al., 1989; Rocha and Albuquerque, 2003) or new items (Thoma et al., 2008). These studies were conducted across a number of different substances, including alcohol (Kramer et al., 1989; Rocha and Albuquerque, 2003; Thoma et al., 2008; Maurage et al., 2011), cannabis (Kloft et al., 2019), and ecstasy/polydrug use (Gallagher et al., 2012). The meta-analysis results indicated that individuals with a history of substance abuse had significantly higher rates of false recognition of unrelated items when compared to healthy comparators. The difference between the groups was medium to large depending on the procedure used.

The results support the hypothesis that different types of intrusion errors (i.e., false recognition/false recall of critical lures and of related and unrelated items) may have distinct underlying neural mechanisms which, in turn, are differently impacted by substance abuse. The hypothesis of distinct neural substrates was first proposed when studies on amnesiacs showed that these individuals had similar decreases in true and related false recognition rates but, paradoxically, presented increases in unrelated false recognition rates (Schacter and Slotnick, 2004). It has since been the subject of further research, for example in the study by Garoff-Eaton et al. (2006), looking into the neural basis of false recognition. These authors found that related and unrelated false recognition of items may be associated with distinct patterns of neural activity, with items related false recognition sharing more specific neural activity with true recognition than with unrelated false recognition. Moreover, the only neural activity uniquely associated with false recognition of unrelated items was registered in brain regions thought to be involved in language processing. According to the above-mentioned authors, these results may be explained by the fact that although no verbalizable shapes were used in the study, participants reported associating verbal labels with these stimuli.

Even though this and similar studies offer important insights into the neural differences between false recognition of related and unrelated items, there is still a lot that is not understood. The results of this review indicate that individuals with a history of substance abuse may have a greater susceptibility to both related and unrelated false recognition, but not to the false recognition of critical lures. Can these results be explained by possible methodological limitations of the studies or are there also significant neural differences between false recognition of related items and false recognition of critical lures? To our knowledge there are no studies looking into this particular question or on how it may relate to the impact of substance abuse on these types of intrusion errors.

### 4.2. Individuals with a history of substance abuse vs. neurological conditions

Only two of the included studies (Welch et al., 1997; Brion et al., 2017) considered a neurological condition as a comparison group, with one focusing on false recognition/false recall (Brion et al., 2017) and the other on provoked confabulation (Welch et al., 1997). Regarding false recognition, Brion et al. (2017), using a continuous recognition task, showed that only patients with Korsakoff Syndrome showed a significantly higher rate of temporal context confusions compared to both alcoholic patients (without that syndrome) and healthy comparators. Similarly, Welch et al. (1997), making use of the Card D of the visual reproduction subtest of the WMS-R, found that only alcoholics with brain damage produced alterations that resembled “drinking vessels”. None of the alcoholics without brain damage or the participants belonging to the other comparison groups (temporal lobe epilepsy, Parkinson, Neurotoxic exposure) produced similar alterations.

### 4.3. Real-world implications of increased susceptibility to false memory formation

Most of the studies reviewed had not the purpose to examine or even discussed potential real-world implications of an increased susceptibility to false memory formation by substance abuse individuals. However, some studies (Reich et al., 2004; Klein et al., 2013) focused not only on the susceptibility to false memory in general, but on the susceptibility to false substance-related memories. Using an adapted DRM paradigm, Reich et al. (2004) found that even though heavy drinkers did not show significantly higher false recognition rates compared to healthy comparators when in a neutral context, they did show a significant increase for target alcohol-expectancy words when in an alcohol-related context (bar). Similarly, Klein et al. (2013) found that patients receiving treatment for alcohol-dependence showed a significant increase in both hit and false alarm rates for alcohol-related words when in comparison with neutral words.

### 4.4. Recommendations for future research

In future studies it would be interesting to understand if the duration and frequency of use are moderators of the impact cannabis and may have an effect on the susceptibility to false memory formation as measured by the DRM paradigm. Likewise, in addition to these factors, it would be important to explore the presence of polydrug use in false memories formation, this because there is a growing interest in recent years in relation to other types of memories (for visual episodic memory; e.g., Binkowska et al., 2021; prospective memory; e.g., Platt et al., 2019).

In turn, it is important to note that we did not find studies on provoked confabulation that included a healthy control group, making it impossible to examine the potential impact of substance abuse on this type of false memory. This gap in the literature signals the need for more research into this question.

We also consider that future studies could investigate how alcoholics with and without brain damage compare to some of the previously mentioned neurological conditions (Section 4.2) on different (and more neutral) measures of provoked confabulation.

Considering that cravings for substance use can often be triggered by memories of past use, we can hypothesize that an increased susceptibility to false memory for substance-related events and/or in a substance-related context could play an important role in relapse. Future research with individuals with a history of substance abuse could also investigate the potential association between false memories, cravings, relapse, as well as previous traumas and unresolved negative emotions (e.g., guilt and shame).

Furthermore, considering the traumatic impact of the COVID-19 pandemic, which possibly resulted in an increase in substance use (Lundahl and Cannoy, 2021; Roberts et al., 2021; Taylor et al., 2021) as a way to mitigate traumatic experiences, it would be important for future research to relate these topics with false memories formation.

Future studies looking into false memory in individuals with a history of substance abuse or with a substance use disorder should consider different well-defined types of false memories (or intrusion errors) and associated measures. Moreover, they should explore the association between increased susceptibility to false memory formation (potentially with regard to substance-related events or in substance-related contexts) and relevant clinical variables.

### 4.5. Limitations

The presented results should be interpreted considering our review's limitations. There was a high level of heterogeneity among the included studies with regards to study design, target population, and procedures for eliciting false memories and therefore how they were measured. This was in part explained by our decision to include all studies with some kind of intrusion error. Even though we consider this option to have enriched the present review by providing a broader overview of the potential impact of substance abuse on memory and false memory formation, it also made it harder to compare the studies' results. Moreover, it makes it impossible to generalize these results to all individuals with a history of substance abuse.

Beyond high heterogeneity, the low number of studies looking into the same substance and considering equivalent false memory procedures/measures also kept us from being able to effectively explore the relative impact of different substances on the susceptibility to false memory formation. Similarly, the existence of only a couple of published studies focusing on provoked confabulations and their lack of a healthy control group kept us from reaching any conclusions, no matter how dubious, regarding the relative susceptibility of individuals with a history of substance abuse to this type of false memory.

It is also important to highlight that some of the studies did not provide all the necessary information that would allow for replication or even for being subject to quality assessment. Many of the studies in the present review did not provide the necessary data to be included in the meta-analysis and only two authors sent the requested missing data when contacted. Thereupon, when doing the analyses according to the outcome, we were only able to consider three of the false memory measures used in the included studies. Moreover, we were not able to complete subgroup analyses according to other relevant variables, such as substance type.

## 5. Conclusion

False memories are a complex topic, made more so by a lack of clarity and consensus in the definitions and considered measures. Although we tried to consider this fact and described each of the constructs included in this review, we do not envision that we, in any way, clarified all the questions and confusion encompassing this subject matter. If anything, the first conclusion that we can make is that there is a need for more scientific discussion going into false memories, what they are and what they are not, what types should be considered, and how they should be elicited and measured. It is also sure that the study of false memories in individuals with a history of substance abuse is very sparse and that, for a better understanding, claims for more acute research. We consider that it would be helpful for future research to continue to include resources written in different languages, not just written in English. This can contribute to a broader understanding of false memories.

Given the high heterogeneity (across several factors) among the included studies in the present review, it is not surprising the observation of some contradictory results. Nonetheless, it is still possible to recognize some trends in this area. Most of the studies using the DRM paradigm and, as such, using false recognition/false recall of critical lures as a measure of false memory, did not find significant differences between substance abuse individuals and healthy comparators. On the other hand, most of the studies considering another type of intrusion error (false recognition/false recall to related and unrelated items) registered significant differences and pointed to increased susceptibility in individuals with a history of substance abuse. Finally, the studies on provoked confabulation were few and their study design did not allow for any conclusions regarding the population of interest.

## Data availability statement

The datasets presented in this study can be found in online repositories. The names of the repository/repositories and accession number(s) can be found in the article/Supplementary material.

## Author contributions

TC, MP, and MD contributed to the conception and design of the study, constant revision, and revised the manuscript critically for relevant intellectual content. TC, ER, JL, and DF conducted the literature search, selection, data extraction, and analysis. TC and JL conducted the assessment of study quality. TA resolved the disagreements. TC, MP, and MD wrote the article, which was critically revised by all other authors. TC, ER, and JL revised the last version of the manuscript. All authors contributed to manuscript revision, read, and approved the submitted version.

## References

[B1] American Psychiatric Association (APA) (2014). Manual de diagnóstico e estatística das perturbações mentais - DSM-5 (5a ed.). Lisboa, Portugal: Climepsi Editores.

[B2] Ardila A. Rosselli M. Strumwasser S. (1991). Neuropsychological deficits in chronic cocaine abusers. Int. J. Neurosci. 57, 73–79. 10.3109/00207459109150348 1938157

[B3] Aromataris E. Munn Z. (Editors) (2017). “Joanna Briggs Institute Reviewer's Manual,” in The Joanna Briggs Institute. Available online at: https://reviewersmanual.joannabriggs.org/ (accessed August 22, 2022).

[B4] Arts N. J. M. Walvoort S. J. W. Kessels R. P. C. (2017). Korsakoff's syndrome: a critical review. Neuropsychiatr. Dis. Treat. 13, 2875–2890. 10.2147/NDT.S130078 29225466 PMC5708199

[B5] Baddeley A. Wilson B. (1988). Frontal amnesia and the dysexecutive syndrome. Brain Cogn. 7, 212–230. 10.1016/0278-2626(88)90031-0 3377900

[B6] Barba G. D. Cipolotti L. Denes G. (1990). Autobiographical memory loss and confabulation in Korsakoff's syndrome: a case report. Cortex. 26, 525–534. 10.1016/S0010-9452(13)80302-4 2081390

[B7] Battisti R. A. Roodenrys S. Johnstone S. J. Respondek C. Hermens D. F. Solowij N. (2010). Chronic use of cannabis and poor neural efficiency in verbal memory ability. Psychopharmacology (Berl). 209, 319–330. 10.1007/s00213-010-1800-4 20217055

[B8] Benson D. F. Djenderedjian A. Miller B. L. Pachana N. A. Chang L. Itti L. . (1996). Neural basis of confabulation. Neurology. 46, 1239–1243. 10.1212/wnl.46.5.1239 8628459

[B9] Berlyne N. (1972). Confabulation. Br. J. Psychiatry. 120, 31–39. 10.1192/bjp.120.554.31 5041518

[B10] Binkowska A. A. Jakubowska N. Gaca M. Galant N. Piotrowska-Cyplik A. Brzezicka A. (2021). Not just a pot: visual episodic memory in cannabis users and polydrug cannabis users: ROC and ERP preliminary investigation. Front. Hum. Neurosci. 15, 1–16. 10.3389/fnhum.2021.677793 34177497 PMC8226271

[B11] Bolla K. I. Brown K. Eldreth D. Tate K. Cadet J. L. (2002). Dose-related neurocognitive effects of marijuana use. Neurology. 59, 1337–1343. 10.1212/01.wnl.0000031422.66442.49 12427880

[B12] Box O. Laing H. Kopelman M. (1999). The evolution of spontaneous confabulation, delusional misidentification and a related delusion in a case of severe head injury. Neurocase. 5, 251–262. 10.1080/13554799908402730

[B13] Brady T. Schacter D. Alvarez G. (2015). The adaptive nature of false memories is revealed by gist-based distortion of true memories. J. Vis. 15, 948. 10.1167/15.12.948

[B14] Brainerd C. J. Reyna V. F. (2002). Fuzzy-trace theory and false memory. Curr. Dir. Psychol. Sci. 11, 164–169. 10.1111/1467-8721.00192

[B15] Brainerd C. J. Reyna V. F. (2004). Fuzzy-trace theory and memory development. Dev. Rev. 24, 396–439. 10.1016/j.dr.2004.08.005 PMC4669979 26644632

[B16] Brion M. de Timary P. Pitel A.-L. Maurage P. (2017). Source memory in Korsakoff syndrome: Disentangling the mechanisms of temporal confusion. Alcohol. Clin. Exp. Res. 41, 596–607. 10.1111/acer.13318 28160301

[B17] Cabeza R. Rao S. M. Wagner A. D. Mayer A. R. Schacter D. L. (2001). Can medial temporal lobe regions distinguish true from false? An event-related functional MRI study of veridical and illusory recognition memory. Proc. Natl. Acad. Sci. U. S. A. 98, 4805–4810. 10.1073/pnas.081082698 11287664 PMC31915

[B18] Cadet J. L. Bisagno V. Milroy C. M. (2014). Neuropathology of substance use disorders. Acta Neuropathol. 127, 91–107. 10.1007/s00401-013-1221-7 24292887 PMC7453825

[B19] Caetano T. Pinho M. S. Ramadas E. Clara C. Areosa T. Dixe M. (2021). Cognitive training effectiveness on memory, executive functioning, and processing speed in individuals with substance use disorders: a systematic review. Front. Psychol. 12. 10.3389/fpsyg.2021.730165 34489833 PMC8418081

[B20] Chanraud S. Leroy C. Martelli C. Kostogianni N. Delain F. Aubin H. J. . (2009). Episodic memory in detoxified alcoholics: contribution of grey matter microstructure alteration. PLoS ONE. 4, e6786. 10.1371/journal.pone.0006786 19707568 PMC2728538

[B21] Cooper J. M. Shanks M. F. Venneri A. (2006). Provoked confabulations in Alzheimer's disease. Neuropsychologia. 44, 1697–1707. 10.1016/j.neuropsychologia.2006.03.029 16697019

[B22] Cuttler C. LaFrance E. M. Stueber A. (2021). Acute effects of high-potency cannabis flower and cannabis concentrates on everyday life memory and decision making. Sci. Rep. 11, 13784. 10.1038/s41598-021-93198-5 34215784 PMC8253757

[B23] Dalla Barba G. (1993). Confabulation: knowledge and recollective experience. Cogn. Neuropsychol. 10, 1–20. 10.1080/02643299308253454

[B24] Dalla Barba G. Guerin B. Brazzarola M. Marangoni S. Barbera C. La Corte V. (2018). The confabulation battery: instructions and international data from normal participants. Neuropsychol. Rehabil. 29, 1625–1636. 10.1080/09602011.2018.1436446 29466921

[B25] Damasio A. R. Graff-Radford N. R. Eslinger P. J. Damasio H. Kassell N. (1985). Amnesia following basal forebrain lesions. Arch. Neurol. 42, 263–271. 10.1001/archneur.1985.04060030081013 3977657

[B26] Deese J. (1959). On the prediction of occurrence of particular verbal intrusions in immediate recall. J. Exp. Psychol. 58, 17–22. 10.1037/h0046671 13664879

[B27] Defranco C. Tarbox A. R. McLaughlin E. J. (1985). Cognitive deficits as a function of years of alcohol abuse. Am. J. Drug Alcohol Abuse 11, 279–293. 10.3109/00952998509016866 4091162

[B28] Dewhurst S. A. Thorley C. Hammond E. R. Ormerod T. C. (2011). Convergent, but not divergent, thinking predicts susceptibility to associative memory illusions. Pers. Individ. Dif. 51, 73–76. 10.1016/j.paid.2011.03.018

[B29] Fisk J. E. Gallagher D. T. Hadjiefthyvoulou F. Montgomery C. (2014). Temporal and visual source memory deficits among ecstasy/polydrug users. Hum. Psychopharmacol. Clin. Exp. 29, 172–182. 10.1002/hup.2385 24446108

[B30] Fox H. C. C. Toplis A. S. S. Turner J. J. D. J. D. Parrott A. C. C. (2001). Auditory verbal learning in drug-free ecstasy polydrug users. Hum. Psychopharmacol. 16, 613–618. 10.1002/hup.344 12404541

[B31] Gallagher D. T. Fisk J. E. Montgomery C. Judge J. Robinson S. J. Taylor P. J. (2012). Effects of ecstasy/polydrug use on memory for associative information. Psychopharmacology (Berl). 222, 579–591. 10.1007/s00213-012-2652-x 22302139

[B32] Garoff-Eaton R. J. Slotnick S. D. Schacter D. L. (2006). Not all false memories are created equal: the neural basis of false recognition. Cereb. Cortex. 16, 1645–1652. 10.1093/cercor/bhj101 16400158

[B33] Gilboa A. Moscovitch M. (2002). “The cognitive neuroscience of confabulation: A review and a model,” in The Handbook of Memory Disorders, Alan, B. A. W., Baddeley, D., and Kopelman, M. D. (John Wiley & Sons, Ltd.). Available online at: https://books.google.pt/books?hl=pt-PT&lr=&id=vprKyQ35MVMC&oi=fnd&pg=PA315&dq=%22The+cognitive+neuroscience+of+confabulation:+A+review+and+a+model%22&ots=hY69Fut3ac&sig=wFs7viihygjQw_waZQFm0uxTRBU&redir_esc=y#v=onepage&q=%22The/cognitive/neuroscience/of/c (accessed July 02, 2021).

[B34] Gillen R. W. Kranzler H. R. Bauer L. O. Burleson J. A. Samarel D. Morrison D. J. (1998). Neuropsychologic findings in cocaine-dependent outpatients. Prog. Neuro-Psychopharmacology Biol. Psychiatry. 22, 1061–1076. 10.1016/S0278-5846(98)00057-8 9829288

[B35] Gudjonsson G. (1987). A parallel form of the gudjonsson suggestibility scale. Br. J. Clin. Psychol. 26, 215–221. 10.1111/j.2044-8260.1987.tb01348.x 3664038

[B36] Gudjonsson G. Hannesdottir K. Agustsson T. Sigurdsson J. Gudmundsdottir A. Pordardottir P. . (2004). The relationship of alcohol withdrawal symptoms to suggestibility and compliance. Psychol. Crime Law 10, 169–177. 10.1080/10683160310001609979

[B37] Gudjonsson G. Hannesdottir K. Petursson H. Tyrfingsson T. (2000). The effects of alcohol withdrawal on memory, confabulation, and suggestibility. Nord. J. Psychiatry 54, 213–220. 10.1080/080394800750019132

[B38] Gündogar D. Demirci S. (2007). Confabulation: a symptom which is intriguing but not adequately known. Turk Psikiyatri Derg. 18, 172–178. 17566883

[B39] Gutchess A. H. Schacter D. L. (2012). The neural correlates of gist-based true and false recognition. Neuroimage. 59, 3418–3426. 10.1016/j.neuroimage.2011.11.078 22155331 PMC3318982

[B40] Hanson K. L. Winward J. L. Schweinsburg A. D. Medina K. L. Brown S. A. Tapert S. F. (2010). Longitudinal study of cognition among adolescent marijuana users over three weeks of abstinence. Addict. Behav. 35, 970–976. 10.1016/j.addbeh.2010.06.012 20621421 PMC2933185

[B41] Henry P. K. Umbricht A. Kleykamp B. A. Vandrey R. Strain E. C. Bigelow G. E. . (2012). Comparison of cognitive performance in methadone maintenance patients with and without current cocaine dependence. Drug Alcohol Depend. 124, 167–171. 10.1016/j.drugalcdep.2011.12.009 22266090 PMC3351542

[B42] Higgins J. Altman D. Sterne J. (2011). “Chapter 8: Assessing risk of bias in included studies,” in Cochrane Handbook for Systematic Reviews of Interventions Version 5.1.0 (updated March 2011), Higgins, J., and Green, S. (eds.). (The Cochrane Collaboration). Available online at: www.handbook.cochrane.org (accessed August 30, 2022).

[B43] Higgins J. P. T. Thompson S. G. Deeks J. J. Altman D. G. (2003). Measuring inconsistency in meta-analyses. BMJ. 327, 557–560. 10.1136/bmj.327.7414.557 12958120 PMC192859

[B44] Hirstein W. (2009). “Memory: Errors, constructive processes, and conscious retrieval,” in Encyclopedia of Consciousness (Amsterdam, Netherlands: Elsevier) p. 1–11. 10.1016/B978-012373873-8.00046-3

[B45] Jacoby L. L. Kelley C. M. Dywan J. (1989). “Memory attributions,” in Varieties of memory and consciousness: Essays in honour of Endel Tulving. (Hillsdale, NJ, US: Lawrence Erlbaum Associates, Inc) p. 391–422.

[B46] Johnson M. K. (1977). “What is being counted none the less?,” in Alcohol and human memory, ed. I. M. Birnbaum & E. S. Parker (Hillsdale, N.J.: Erlbaum).

[B47] Johnson M. K. Bransford J. D. Solomon S. K. (1971). Short reports memory for tacit implications of sentences. J. Exp. Psychol. 98, 203–205. 10.1037/h0034290

[B48] Johnson M. K. Nolde S. F. Mather M. Kounios J. Schacter D. L. Curran T. (1997). The similarity of brain activity associated with true and false recognition memory depends on test format. Psychol. Sci. 8, 250–257. 10.1111/j.1467-9280.1997.tb00421.x

[B49] Johnson M. K. Raye C. L. (1981). Reality monitoring. Psychol. Rev. 88, 67–85. 10.1037/0033-295X.88.1.67

[B50] Klein A. A. Nelson L. M. Anker J. J. (2013). Attention and recognition memory bias for alcohol-related stimuli among alcohol-dependent patients attending residential treatment. Addict. Behav. 38, 1687–1690. 10.1016/j.addbeh.2012.10.006 23254219

[B51] Kloft L. Monds L. A. Blokland A. Ramaekers J. G. Otgaar H. (2021). Hazy memories in the courtroom: a review of alcohol and other drug effects on false memory and suggestibility. Neurosci. Biobehav. Rev. 124, 291–307. 10.1016/j.neubiorev.2021.02.012 33587958

[B52] Kloft L. Otgaar H. Blokland A. Garbaciak A. Monds L. A. Ramaekers J. G. (2019). False memory formation in cannabis users: a field study. Psychopharmacology (Berl). 236, 3439–3450. 10.1007/s00213-019-05309-w 31250074 PMC6892757

[B53] Kopelman M. (1999). Varieties of false memory. Cogn. Neuropsychol. 16, 197–214. 10.1080/026432999380762

[B54] Kopelman M. Thomson A. Guerrini I. Marshall E. (2009). The Korsakoff syndrome: Clinical aspects, psychology and treatment. Alcohol Alcohol. 44, 148–154. 10.1093/alcalc/agn118 19151162

[B55] Kopelman M. D. (1987). Two types of confabulation. J. Neurol. Neurosurg. Psychiatry 50, 1482–1487. 10.1136/jnnp.50.11.1482 3694207 PMC1032561

[B56] Kopelman M. D. Ng N. Van Den Brouke O. (1997). Confabulation extending across episodic, personal, and general semantic memory. Cogn. Neuropsychol. 14, 683–712. 10.1080/026432997381411

[B57] Korsakoff S S. (1887). “Disturbance of psychic function in alcoholic paralysis and its relation to the disturbance of the psychic sphere in multiple neuritis of non-alcoholic origin,” in The Wernicke?Korsakoff Syndrome. eds Victor, M., Adams, R, D., Collins, G, H. (Oxford: Blackwell).

[B58] Korsakoff S S. (1889a). Psychic disorder in conjunction with peripheral neuritis. Neurology. 5:394–406. 14383944 10.1212/wnl.5.6.394

[B59] Korsakoff S S. (1889b). Etude medico-psychologique sur une forme des maladies de la me moire. Revue Philosophie 20:501–30.

[B60] Kramer J. H. Blusewicz M. J. Preston K. A. (1989). The premature aging hypothesis: old before its time? J. Consult. Clin. Psychol. 57, 257–262. 10.1037/0022-006X.57.2.257 2708614

[B61] Lundahl L. H. Cannoy C. (2021). COVID-19 and Substance Use in Adolescents. Pediatr. Clin. North Am. 68, 977–990. 10.1016/j.pcl.2021.05.005 34538307 PMC8445753

[B62] Maurage P. Callot C. Chang B. Philippot P. Rombaux P. de Timary P. (2011). Olfactory impairment is correlated with confabulation in alcoholism: towards a multimodal testing of orbitofrontal cortex. PLoS ONE. 6, e23190. 10.1371/journal.pone.0023190 21858026 PMC3155545

[B63] McDermott K. B. Watson J. M. (2001). The rise and fall of false recall: the impact of presentation duration. J. Mem. Lang. 45, 160–176. 10.1006/jmla.2000.2771

[B64] Meier M. H. Caspi A. Ambler A. Harrington H. L. Houts R. Keefe R. S. E. . (2012). Persistent cannabis users show neuropsychological decline from childhood to midlife. Proc. Natl. Acad. Sci. U. S. A. 109, E2657-E2664. 10.1073/pnas.1206820109 22927402 PMC3479587

[B65] Moscovitch M. Melo B. (1997). Strategic retrieval and the frontal lobes: evidence from confabulation and amnesia. Neuropsychologia. 35, 1017–1034. 10.1016/S0028-3932(97)00028-6 9226662

[B66] Nahum L. Bouzerda-Wahlen A. Guggisberg A. Ptak R. Schnider A. (2012). Forms of confabulation: dissociations and associations. Neuropsychologia. 50, 2524–2534. 10.1016/j.neuropsychologia.2012.06.026 22781813

[B67] Nedjam Z. Barba G. D. Pillon B. (2000). Confabulation in a patient with fronto-temporal dementia and a patient with Alzheimer's disease. Cortex. 36, 561–577. 10.1016/S0010-9452(08)70538-0 11059455

[B68] Pardilla-Delgado E. Payne J. D. (2017). The Deese-Roediger-Mcdermott (DRM) task: A simple cognitive paradigm to investigate false memories in the laboratory. J. Vis. Exp. 2017, 1–10. 10.3791/54793 28190038 PMC5407674

[B69] Pierce B. H. Schacter D. L. Sullivan A. L. Budson A. E. (2005). Comparing source-based and gist-based false recognition in aging and Alzheimer's disease. Neuropsychology. 19, 411–419. 10.1037/0894-4105.19.4.411 16060815

[B70] Platt B. O'Driscoll C. Curran V. H. Rendell P. G. Kamboj S. K. (2019). The effects of licit and illicit recreational drugs on prospective memory: a meta-analytic review. Psychopharmacology (Berl). 236, 1131–1143. 10.1007/s00213-019-05245-9 31093722 PMC6591206

[B71] Pope H. G. Gruber A. J. Hudson J. I. Huestis M. A. Yurgelun-Todd D. (2001). Neuropsychological performance in long-term cannabis users. Arch. Gen. Psychiatry 58, 909–915. 10.1001/archpsyc.58.10.909 11576028

[B72] Reich R. R. Goldman M. S. Noll J. A. (2004). Using the false memory paradigm to test two key elements of alcohol expectancy theory. Exp. Clin. Psychopharmacol. 12, 102–110. 10.1037/1064-1297.12.2.102 15122954

[B73] Rendell P. G. Mazur M. Henry J. D. (2009). Prospective memory impairment in former users of methamphetamine. Psychopharmacology (Berl). 203, 609–616. 10.1007/s00213-008-1408-0 19037633

[B74] Rensen Y. C. M. Kessels R. P. C. Migo E. M. Wester A. J. Eling P. A. T. M. Kopelman M. D. (2017). Personal semantic and episodic autobiographical memories in Korsakoff syndrome: a comparison of interview methods. J. Clin. Exp. Neuropsychol. 39, 534–546. 10.1080/13803395.2016.1248811 27829317

[B75] Rensen Y. C. M. Oosterman J. M. Van Damme J. E. Griekspoor S. I. A. Wester A. J. Kopelman M. D. . (2015). Assessment of confabulation in patients with alcohol-related cognitive disorders: the Nijmegen-Venray confabulation list (nvcl-20). Clin. Neuropsychol. 29, 804–823. 10.1080/13854046.2015.1084377 26360957

[B76] Riba J. Valle M. Sampedro F. Rodríguez-Pujadas A. Martínez-Horta S. Kulisevsky J. . (2015). Telling true from false: Cannabis users show increased susceptibility to false memories. Mol. Psychiatry 20, 772–777. 10.1038/mp.2015.36 25824306 PMC4441258

[B77] Roberts A. Rogers J. Mason R. Siriwardena A. N. Hogue T. Whitley G. A. . (2021). Alcohol and other substance use during the COVID-19 pandemic: a systematic review. Drug Alcohol Depend. 229, 109150. 10.1016/j.drugalcdep.2021.109150 34749198 PMC8559994

[B78] Rocha A. A. M. Albuquerque P. B. (2003). Ilusões de memória em alcoólicos. Psicol. Teor. Investig. e Prática 2, 269–288.

[B79] Roediger H. L. McDermott K. B. (1995). Creating false memories: remembering words not presented in lists. J. Exp. Psychol. Learn. Mem. Cogn. 21, 803–814. 10.1037/0278-7393.21.4.80321331837

[B80] Roediger H. L. Watson J. M. McDermott K. B. Gallo D. A. (2001). Factors that determine false recall: a multiple regression analysis. Psychon. Bull. Rev. 8, 385–407. 10.3758/BF03196177 11700893

[B81] Schacter D. L. Guerin S. A. St Jacques P. L. (2011). Memory distortion: an adaptive perspective. Trends Cogn. Sci. 15, 467–474. 10.1016/j.tics.2011.08.004 21908231 PMC3183109

[B82] Schacter D. L. Slotnick S. D. (2004). The cognitive neuroscience of memory distortion. Neuron 44, 149–160. 10.1016/j.neuron.2004.08.017 15450167

[B83] Schilt T. Win M. M. L. d. Jager G. Koeter M. W. Ramsey N. F. Schmand B. . (2008). Specific effects of ecstasy and other illicit drugs on cognition in poly-substance users. Psychol. Med. 38, 1309–1317. 10.1017/S0033291707002140 17988417

[B84] Schnider A. (2003). Spontaneous confabulation and the adaptation of thought to ongoing reality. Nat. Rev. Neurosci. 4, 662–671. 10.1038/nrn1179 12894241

[B85] Schnider A. Ptak R. (1999). Spontaneous confabulators fail to suppress currently irrelevant memory traces. Nat. Neurosci. 2, 677–681. 10.1038/10236 10404203

[B86] Schnider A. Von Däniken C. Gutbrod K. (1996). The mechanisms of spontaneous and provoked confabulations. Brain. 119, 1365–1375. 10.1093/brain/119.4.1365 8813298

[B87] Scott J. C. Woods S. P. Matt G. E. Meyer R. A. Heaton R. K. Atkinson J. H. . (2007). Neurocognitive effects of methamphetamine: a critical review and meta-analysis. Neuropsychol. Rev. 17, 275–297. 10.1007/s11065-007-9031-0 17694436

[B88] Steffens M. C. Mecklenbräuker S. (2007). False memories: phenomena, theories, and implications. J. Psychol. 215, 12–24. 10.1027/0044-3409.215.1.12

[B89] Straube B. (2012). An overview of the neuro-cognitive processes involved in the encoding, consolidation, and retrieval of true and false memories. Behav. Brain Funct. 8, 1–10. 10.1186/1744-9081-8-35 22827854 PMC3411412

[B90] Strickland T. L. Mena I. Villanueva-Meyer J. Miller B. L. Cummings J. Mehringer C. M. . (1993). Cerebral perfusion and neuropsychological consequences of chronic cocaine use. J. Neuropsychiatry Clin. Neurosci. 5, 419–427. 10.1176/jnp.5.4.419 8286941

[B91] Sullivan E. Pfefferbaum A. (2013). Neuropsychology and neuroimaging studies in alcohol-dependence. Rev. Neuropsychol. 5, 187–199. 10.3917/rne.053.0187

[B92] Taylor S. Paluszek M. M. Rachor G. S. McKay D. Asmundson G. J. G. (2021). Substance use and abuse, COVID-19-related distress, and disregard for social distancing: a network analysis. Addict. Behav. 114, 106754. 10.1016/j.addbeh.2020.106754 33310690 PMC8164919

[B93] Thoma P. Johann K. Wähner A. Juckel G. Daum I. (2008). Recollective experience in alcohol dependence: a laboratory study. Addiction 103, 1969–1978. 10.1111/j.1360-0443.2008.02374.x 19469740

[B94] Thomasius R. Zapletalova P. Petersen K. Buchert R. Andresen B. Wartberg L. . (2006). Mood, cognition and serotonin transporter availability in current and former ecstasy (MDMA) users: the longitudinal perspective. J. Psychopharmacol. 20, 211–225. 10.1177/0269881106059486 16510479

[B95] Turner M. S. Cipolotti L. Yousry T. A. Shallice T. (2008). Confabulation: Damage to a specific inferior medial prefrontal system. Cortex 44, 637–648. 10.1016/j.cortex.2007.01.002 18472034

[B96] Vonmoos M. Hulka L. M. Preller K. H. Jenni D. Baumgartner M. R. Stohler R. . (2013). Cognitive dysfunctions in recreational and dependent cocaine users: Role of attention-deficit hyperactivity disorder, craving and early age at onset. Br. J. Psychiatry 203, 35–43. 10.1192/bjp.bp.112.118091 23703315

[B97] Welch L. W. W. Nimmerrichter A. Gilliland R. King D. E. E. Martin P. R. R. (1997). “Wineglass” confabulations among brain-damaged alcoholics on the Wechsler Memory Scale-Revised visual reproduction subtest. Cortex. 33, 543–551. 10.1016/S0010-9452(08)70235-1 9339334

[B98] Woicik P. A. Moeller S. J. Alia-Klein N. Maloney T. Lukasik T. M. Yeliosof O. . (2009). The neuropsychology of cocaine addiction: Recent cocaine use masks impairment. Neuropsychopharmacology 34, 1112–1122. 10.1038/npp.2008.60 18496524 PMC2667096

